# Superchaotropic Stabilization of Monomeric Protein States

**DOI:** 10.1021/acs.biomac.5c00944

**Published:** 2025-09-01

**Authors:** Ben Tin Yan Wong, Lichun Zhang, Thomas Chun Yip Wong, Chun Ngo Yau, Adrian Jun Chu, Tsz Fung Tsang, Joshua Jing Xi Li, Xiao Yang, Hei Ming Lai

**Affiliations:** 1 Department of Chemical Pathology, Faculty of Medicine, 26451 The Chinese University of Hong Kong, Shatin, Hong Kong SAR, China; 2 Illumos Limited, Hong Kong Science and Technology Park, Shatin, Hong Kong SAR, China; 3 Li Ka Shing Institute of Health Sciences, Faculty of Medicine, The Chinese University of Hong Kong, Shatin, Hong Kong SAR, China; 4 Department of Psychiatry, Faculty of Medicine, 26451 The Chinese University of Hong Kong, Shatin, Hong Kong SAR, China; 5 Department of Microbiology, Faculty of Medicine, 26451 The Chinese University of Hong Kong, Shatin, Hong Kong SAR China; 6 Department of Pathology, School of Clinical Medicine, The University of Hong Kong, Queen Mary Hospital, Pok Fu Lam, Hong Kong SAR, China

## Abstract

Chaotropes are long known to destabilize protein assemblies and folding. We report that a boron cluster ion, as a weakly coordinating superchaotrope, can paradoxically stabilize protein folding even under extended thermal stresses while broadly inhibiting specific and nonspecific protein–protein interactions at millimolar concentrations for multiple proteins. Thermodynamic and kinetic investigations suggest that the boron cluster ion reduced the association rates of protein association and rendered protein-associative interactions entropically unfavorable. The preliminary utility of this phenomenon is demonstrated by the preservation of protein functions within complex mixtures stored in ambient, uncontrolled conditions, boosting their shelf life and stability against aggregation.

## Graphical abstract

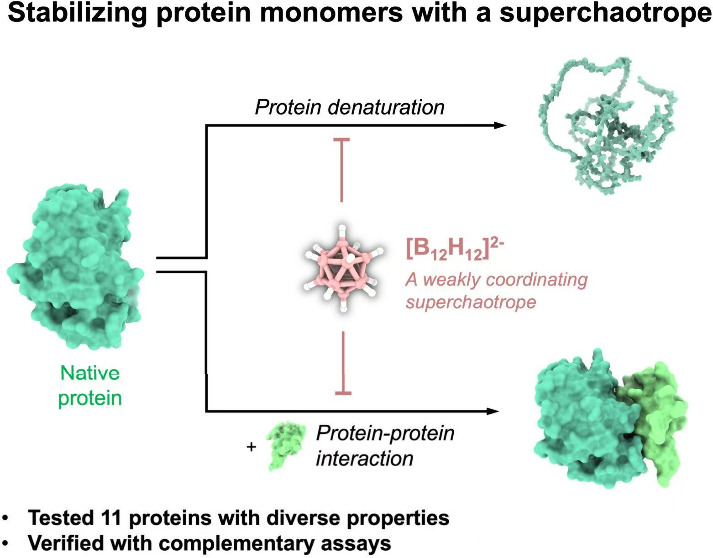

## Introduction

Proteins and their mutual interactions form the functional cornerstones of life and their associated applications. The precise and predictable modulation of protein functional assemblies is essential for modern therapeutics, biotechnologies, and industrial applications. However, the unique three-dimensional conformations that confer proteins their highly specific functions also make them susceptible to destruction due to the near-infinite alternative conformations their primary sequences can explore.^1,2^ Consequently, substantial research has focused on developing protein stabilization strategies,^3^ including directed evolution of resilient mutants,^4^ chemical modifications,^5–7^ and cosolvent use^8–11^ leading to preferential protein hydration. The cosolvent or cosolute approach is desirable as it avoids covalent modification and is readily implementable by virtue of a single additive while offering general applicability based on the fundamental principles of statistical mechanics.^12^

Certain cosolutes are known to stabilize protein conformations while others destabilize them. Figure 1a illustrates our current understanding of these mechanisms. Osmolytes or kosmotropes such as polyethylene glycols (PEGs), trehalose, and trimethylamine oxide (TMAO) are preferentially excluded from macromolecular surfaces, leading to preferential hydration and stabilization of native conformations, where hydrophilic residues remain exposed while hydrophobic residues stay buried.^13–18^ Conversely, chaotropes destabilize proteins by preferentially associating with surfaces and backbones. Recent thermodynamic analyses suggest destabilizing cosolutes—both chaotropes and certain osmolytes in specific contexts—directly interact with protein surfaces to favor unfolded states through enthalpically driven processes.^19–21^ These direct interactions stabilize protein states even when hydrophobic residues are exposed, shifting the equilibrium toward unfolding, as illustrated in Figure 1b. The direct destabilizing cosolute–protein interactions compensate for the enthalpic requirements in breaking intramolecular bonds, while the protein gains conformational entropy as a driving force toward unfolding and denaturation. Additional theoretical details have been provided in Supplementary Note 1.

**Figure 1:**
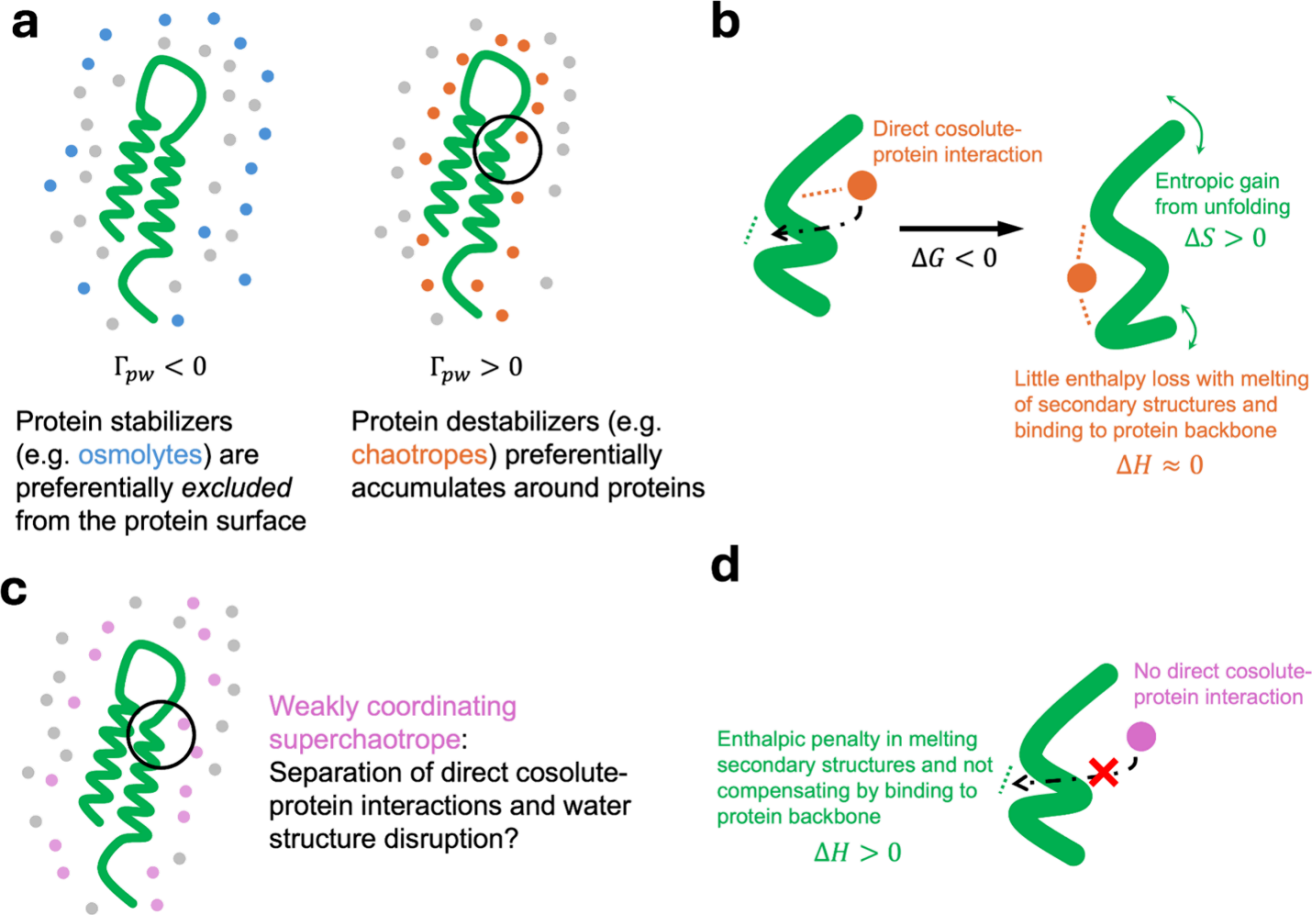
Effects of cosolutes on protein stability. (a) Protein stabilizers and denaturants are known to be preferentially excluded and accumulated around proteins, respectively, reflected from statistical mechanical inference of experimental data on the preferential interaction coefficient between protein (p, in green) and water (w, in gray), Γ_pw_. (b) It is thought that direct interactions between chaotropes, as circled in panel (a), promote protein unfolding. (c) The case of an additive that possesses water structure-breaking properties of chaotrope but does not accumulate around proteins, i.e., that of a weakly coordinating superchaotrope, is unexplored. (d) Based on current theory, a weakly coordinating superchaotrope can paradoxically be protein-stabilizing, as opposed to conventional assumptions.

Beyond preferential association or exclusion with biomacromolecules, cosolutes are also thought to influence protein stability by affecting hydrogen bonding networks and water structuring distant from macromolecular surfaces. According to the Hofmeister series, kosmotropes enhance hydrogen bonds in the solvent, promoting protein stabilization, while chaotropes disrupt these networks, leading to destabilization. However, the physical mechanism of how a change in water bonding affects protein stability is less thoroughly investigated experimentally.^22^

Interestingly, cosolutes generally recognized as protein-stabilizing can lead to denaturation if they directly interact with proteins through enthalpic means,^23–25^ even if they promote water structure formation. We thus questioned what would happen when a highly water-soluble cosolute is simultaneously weakly coordinating and water structure disrupting (Figure 1c)—would it stabilize or destabilize proteins? A weakly coordinating agent would minimize destabilizing direct cosolute–protein interactions, as illustrated in Figure 1d, while high water solubility would reduce effective interactions between proteins and the cosolute. Meanwhile, a superchaotropic agent would significantly disrupt water structure even at low concentrations. However, to date, no known cosolute has segregated these properties.

To experimentally investigate our question, we searched for conjugate bases of superacids and weakly coordinating anions^26^ used for the crystallization of highly electrophilic species for X-ray crystallography and superchaotropic species that can reduce the formation of hydrogen bonds, characterized by highly negative ΔHB values as described by Marcus^27^ and derived by Assaf et al.^28^ Based on this reductionist approach, we identified the dodecahydroclosododecaborate ion ([B_12_H_12_]^2–^) as a chemically and biologically inert, weakly coordinating superchaotrope that would be an interesting case for investigations. Indeed, [B_12_H_12_]^2–^ has been found to be weakly coordinating in two cases with minimal disturbance to myoglobin^29^ and bovine serum albumin (BSA).^30^

## Materials and Methods

### Materials

Sodium dodecahydro-*closo*-dodecaborate was procured from Katchem (catalog no. 257) and used without further purification.

### Dynamic Light Scattering

The hydrodynamic diameters (particle sizes) of the proteins dissolved in 1× PBS and various concentrations of Na_2_B_12_H_12_ were measured by the dynamic light scattering method using a Zetasizer (Malvern Pananalytical, ZS-90). Particle size was represented by z-average, and the polydispersity index (PdI) was calculated based on at least three independent measurements (*N* = 3).

### Microscale Thermophoresis (MST)

MST analysis was performed on the Monolith instrument developed by NanoTemper Technologies GmbH (Munich, Germany), and Monolith LabelFree Premium Capillaries were used. To induce fluorescence in Alexa Fluor 594 (AF594)-labeled samples, red LED light was utilized. The MST power was set to 40%, and the LED power was set to the auto mode.

To study protein stability in the presence of [B_12_H_12_]^2–^, 222 nM of AF594-labeled rabbit IgG (homemade and purified) was incubated with varying concentrations of [B_12_H_12_]^2–^ in PBS for 10 min at room temperature.

To study protein–protein interactions in the presence of [B_12_H_12_]^2–^, 200 nM of AF594-labeled donkey antirabbit Fab fragment (Jackson Immunoresearch, 711-587-003) was incubated with varying concentrations of rabbit IgG (Invitrogen, 02-6102) in the presence or absence of [B_12_H_12_]^2–^ in PBS for 10 min at room temperature.

Subsequently, the sample solutions were serially diluted and loaded into capillaries for analysis. Sixteen capillaries were simultaneously analyzed using MST. The MST signal of each sample was recorded over time, both before and after activation of the IR-laser heating. The IR laser was turned off after 5 s. The normalized fluorescence (*F*_norm_) was used as the MST signal. The MST instrument includes temperature control, allowing measurements to be taken within a temperature range of 22 to 45 °C. The detection temperature was set at 25 °C unless otherwise specified. For data analysis, the MO. Affinity Analysis software from Nanotemper Technologies (Germany) was used to determine the dissociation constant (*K*_d_).

### Circular Dichroism (CD)

Circular dichroism experiments were conducted on a CD spectrophotometer (J-1500). The CD spectra were recorded over a wavelength range of 260–200 nm with “continuous” scanning mode at 100 nm/min scan speed with a 1 nm resolution and D.I.T. of 4 s. BSA and mIgG were used as models in the experiments.

### Mouse Anti-His_6_ Tag ELISA Assay in the Presence of [B_12_H_12_]^2–^

Mouse His_6_-tag ELISA assay kit from Genscript (L00436) was used as received. 50 μL of the provided monoclonal anti-His_6_ tag antibody was diluted 25 μL of the Na_2_B_12_H_12_ solution of various concentrations and added to the His_6_-tagged protein-coated wells. Subsequent incubation, washings, addition of detection reagent (horseradish peroxidase-conjugated antimouse antibodies), and signal generation were performed as per the assay manual. Quantification was performed with a Viktor3 spectrophotometer at 450 nm.

To verify that His_6_-tagged proteins were not stripped from the wells by Na_2_B_12_H_12_, the provided coated wells were incubated with 75 μL of 0.25 M Na_2_B_12_H_12_ for 30 min at room temperature. The Na_2_B_12_H_12_-treated wells were then assayed as usual with untreated wells, which did not result in a signal difference by the end of the assay.

### Isothermal Titration Calorimetry (ITC)

The titration experiments were conducted on a Malvern Panalytical MicroCal PEAQ-ITC. All injections were performed in high-feedback mode with a stirring speed of 750 rpm at 25 °C. Trypsin and ovomucoid were used as a protein–protein interaction model throughout the whole experiment. 0.15 mM ovomucoid was titrated against 1.8 mM trypsin in the same buffer. The initial delay time is 60 s. The initial injection was 0.4 μL, followed by 26 injections, each with a volume of 1.4 μL subsequently injected every 150 s. All of the ITC results were fitted with the “One set of sites” model. The stoichiometric number (*N*) and the dissociation constant (*K*_d_) and enthalpy change (Δ*H*) were obtained from Malvern ITC software, and Δ*G* and *T*Δ*S* were calculated from the equation: Δ*G* = – *RT* ln­(*K*_a_) and *T*Δ*S* = Δ*H* – Δ*G* respectively. *R* is the molar gas constant, and *T* is the absolute temperature used for the experiment. Raw calorimetric data were analyzed using MicroCal PEAQ-ITC analysis software v1.1.0.1262, and final figures were made using GraphPad Prism 8.0. The kinITC-ETC method^38^ was implemented by preprocessing raw data from ITC measurement in Affinimeter software followed by curve fitting by custom MATLAB code to obtain kinetic rate constants k_on and k_off. For each injection, the baseline was corrected by removing a fitted smoothing spline of a value greater than 0 from raw data. Then, the baseline corrected data from the minimum to the end of injection was fitted to an exponential curve with characteristic time constant. The strategy to locate the equilibrium time was integrating the area of the fitted curve and selecting the area fraction as a parameter that yields an equilibration time when the curve has plateaued. After trials of varying area fractions, 0.997 of the total area was chosen to determine the equilibration time. Then, the equilibration time curve (ETC) was plotted against the molar ratio of titrant (content in injection syringe) to titrand (content in the measurement cell) in the ITC experiment. The curve was fitted to the equation with fitting parameters *a*, *A*_tot_, and *k*_on_ for PBS to determine machine-related constant *a* and fit ETC for Na_2_[B_12_H_12_] with only *A*_tot_ and *k*_on_. The *k*_off_ is calculated by the relation *K*_d_ = *k*_off_/*k*_on_, and the equilibration time is calculated by the equation *t*_eq_ = α [ *k*_on_ *A*_tot_ (1 + *K*_d_/*A*_tot_ + *s*)^2^ – 4*s* ]^−1^ + *a*, where *a* = α τ_ITC_ + τ_inj_ + τ_mix_ is a constant related to ITC machine settings and properties and α is a pragmatic approximation coefficient tuned to 4.5 to relate the equilibration time and characteristic time.

### Biolayer Interferometry (BLI)

Biolayer interferometric assays were performed on an Octet RED instrument (ForteBio, Inc., model RED96) to obtain kinetic information. Purified monoclonal mouse calbindin D28k IgGs (Synaptic systems, 214-011) were first loaded onto immobilized anti-mIgG Fc capture probes (Sartorius, Inc., AMC2 Biosensors) in 1× kinetic buffer (1× KB) consisting of 1× PBS (pH 7.4), 0.1% BSA and 0.02% Tween-20 for 600 s. The probes were then incubated with calbindin D28K protein (Synaptic systems, 214-0P) for 600 s in 1× KB in the presence or absence of 0.25 M Na_2_B_12_H_12_, followed by 900s in 1× KB for dissociation. The temperature was controlled at 25 °C throughout the whole experiment. The data were then analyzed with ForteBio Data Analysis 11.0 software fitted to a 1:1 binding model to determine the association rate constant (*k*_on_), dissociation rate constant (*k*_off_), and binding affinity constant (*K*_D_).

### Gelatin Gelation in the Presence of [B_12_H_12_]^2–^

2% w/v gelatin (Sigma, G2500) stock solutions were prepared by gentle heating using a microwave until all solids had been dissolved. 2.5 M stocks of Na_2_B_12_H_12_ or NaCl were then added at 1:10 v/v ratios to the gel solutions. A small crystal of acid fuchsin was added to enhance the visibility during the gelation test. The gelatin solution was refrigerated at 4 °C for 10 min. Gelation was confirmed by inverting the tubes under gravity.

### Thermal Denaturation of Fluorescent Proteins

1 μL fluorescent proteins (1 mg/mL each. mCherry, Abcam, ab199750; GFP, Abcam, ab134853; Y1-BFP, Abcam, ab269139) were diluted in 10 μL of 1× PBS, followed by the addition of 1 μL of 0.5 M Na_2_B_12_H_12_, 0.5 M SDS stock solution, or water in PCR tubes. The mixtures were then incubated in a thermocycler kept at 55 °C for up to 72 h. Fluorescence intensities were observed at various time points under UV light at 365 nm wavelength and photographs taken with a smartphone camera (Apple Inc., iPhone 11 Pro).

### Amyloid Beta (1–42) Aggregation Assay in the Presence of [B_12_H_12_]^2–^

Thioflavin T (MedChemExpress, HY-D0218) and amyloid beta (1–42) peptide (Aβ_1–42_) (Anaspec, AS-24224) were dissolved in 1× PBS and combined at concentrations of 20 and 40 μM, respectively. A 50 μL aliquot of the ThT and Aβ_1–42_ mixture was then added to 50 μL of [B_12_H_12_]^2–^ or 1× PBS with a series of varying concentrations, resulting in final concentrations of 10 μM for ThT and 20 μM for Aβ42. ThT fluorescence was measured using a Tecan Spark Microplate reader in a Greiner 96-well flat transparent plate at 37 °C. The plate was shaken for 20 s before each measurement, which was taken every 20 min over a 5 h period, with excitation at 450 nm and emission at 490 nm. *N* = 3 replicates were performed.

### Egg White Thermal Coagulative Aggregation in the Presence of [B_12_H_12_]^2–^

Hen or quail eggs were obtained from a local market and were freshly cracked. The egg whites were separated from the yolk and diluted with 0.9% (w/v) NaCl at 1:1 v/v ratio. The diluted egg white mixture was collected in two syringes with Luer locks, which were then connected to a three-way syringe connector. All bubbles were carefully eliminated, and the valve was set to open the flow between the syringes but not out. The fluids were then pumped alternately between the syringes until the diluted egg whites were thoroughly mixed and sheared. 0.5 M stocks of Na_2_B_12_H_12_ or NaCl were then added in 1:10 v/v ratio to the diluted egg whites and mixed in a similar manner. The egg whites were then cooked in a water bath or in PCR tubes using a thermocycler.

### Long-Term Dormancy and Reactivation of Histochemical Reagents

Three staining solutions were prepared for the experiment as follows:

- 3 μg of anti-Na^+^/K^+^-ATPase antibody (Abclonal, A12405), 3 μg of AlexaFluor 488-labeled Donkey antirabbit Fab fragment (Jackson Immunoresearch, 711-547-003), and 1 μL of 1:1 v/v premixed DAPI dilactate (43.6 mM stock, Invitrogen, D3571) and sulfobutylether-β-cyclodextrin solution (SBEβCD, 0.1 M stock, Cyclolab, 47), in 100 μL 1× PBS with 0.25 M [B_12_H_12_]^2–^ and 0.02% w/v NaN_3_
- 3 μg of anti-Vimentin antibody (Abclonal, A19607), 3 μg of AlexaFluor 488-labeled Donkey antirabbit Fab fragment (Jackson Immunoresearch, 711-547-003), 5 μg of DyLight 649-labeled *Lycopersicon esculentum* lectin (Vector Laboratories, DL-1178-1), and 1 μL of 1:1 v/v premixed DAPI dilactate (43.6 mM stock) and SBEβCD (0.1 M stock), in 100 μL 1× PBS with 0.25 M [B_12_H_12_]^2–^ and 0.02% w/v NaN_3_
- 3 μg of anti-CD45 antibody (Abclonal, A2115), 3 μg of AlexaFluor 647-labeled Donkey antirabbit Fab fragment (Jackson Immunoresearch, 711-607-003), 5 μg of DyLight 594-labeled *Griffonia simplicifolia* lectin I (Vector Laboratories, DL-1207-.5), and 1 μL of 1:1 v/v premixed DAPI dilactate (43.6 mM stock) and SBEβCD (0.1 M stock), in 100 μL 1× PBS with 0.25 M [B_12_H_12_]^2–^ and 0.02% w/v NaN_3_

The addition of SBEβCD was used to complex the positively charged DAPI dye to prevent its precipitation with [B_12_H_12_]^2–^. SBECD does not bind to [B_12_H_12_]^2–^ due to its small size of the β-cyclodextrin backbone (*K*_d_ = 0.1 M^55^) and associated negative charge. The solutions were then stored in the dark at ambient temperature (15–25 °C) for 60 days. After incubation, the mixtures were buffer-exchanged into 1× PBS using ultracentrifugal filters and applied to stain mouse kidney and human colon tissues. The tissues used were 4% paraformaldehyde-fixed, cut into 1 mm-thick slices, washed in 1× PBS, dehydrated in 50% v/v methanol/H_2_O, 100% methanol, and repeated in 100% methanol for 15 min each at room temperature and delipidated in 2:1 v/v CH_2_Cl_2_/methanol overnight at room temperature. The tissues were then rehydrated by reversing the methanol dehydration steps, washed adequately in 1× PBS, and stained in aged staining solutions. As a control, freshly prepared solutions with identical formulas were also applied to the same batch of tissue duplicates. The staining was allowed to proceed at room temperature for 1 day and washed for 1 day in 1 mL of 1× PBS. The tissues were then dehydrated with graded methanol, cleared in a 2:1 v/v mixture of benzyl alcohol and benzyl benzoate, and imaged using a Leica SP8 confocal microscope equipped with a white light laser and a ×40 oil-immersion objective (HC PL APO 40*x*/1.30 Oil CS2). The fluorophores were excited at their corresponding emission peaks for imaging with identical laser power and gains for the paired specimens. No gamma adjustments were performed for the images.

## Results

### Superchaotropic Ion [B_12_H_12_]^2–^ Does Not Promote Protein Denaturation

We first investigated whether the superchaotropic ion [B_12_H_12_]^2–^ would denature proteins. We first utilized dynamic light scattering (DLS) to test whether there is a change in the molecular hydrodynamic radii distribution in the presence of increasing [B_12_H_12_]^2–^ concentrations. The formation of larger species would be indicative of protein aggregation.^31^ Figure 2a and Supplementary Figure 1 show the DLS results when 0 to 0.5 M [B_12_H_12_]^2–^ was incubated with immunoglobulin G (IgG) at 37 °C. IgG is a multidomain protein that is prone to aggregation even when partially unfolded.^32^ At all concentrations of [B_12_H_12_]^2–^ tested, the hydrodynamic radii remained essentially unchanged, suggesting the absence of protein aggregation, which would indicate denaturation.

**Figure 2:**
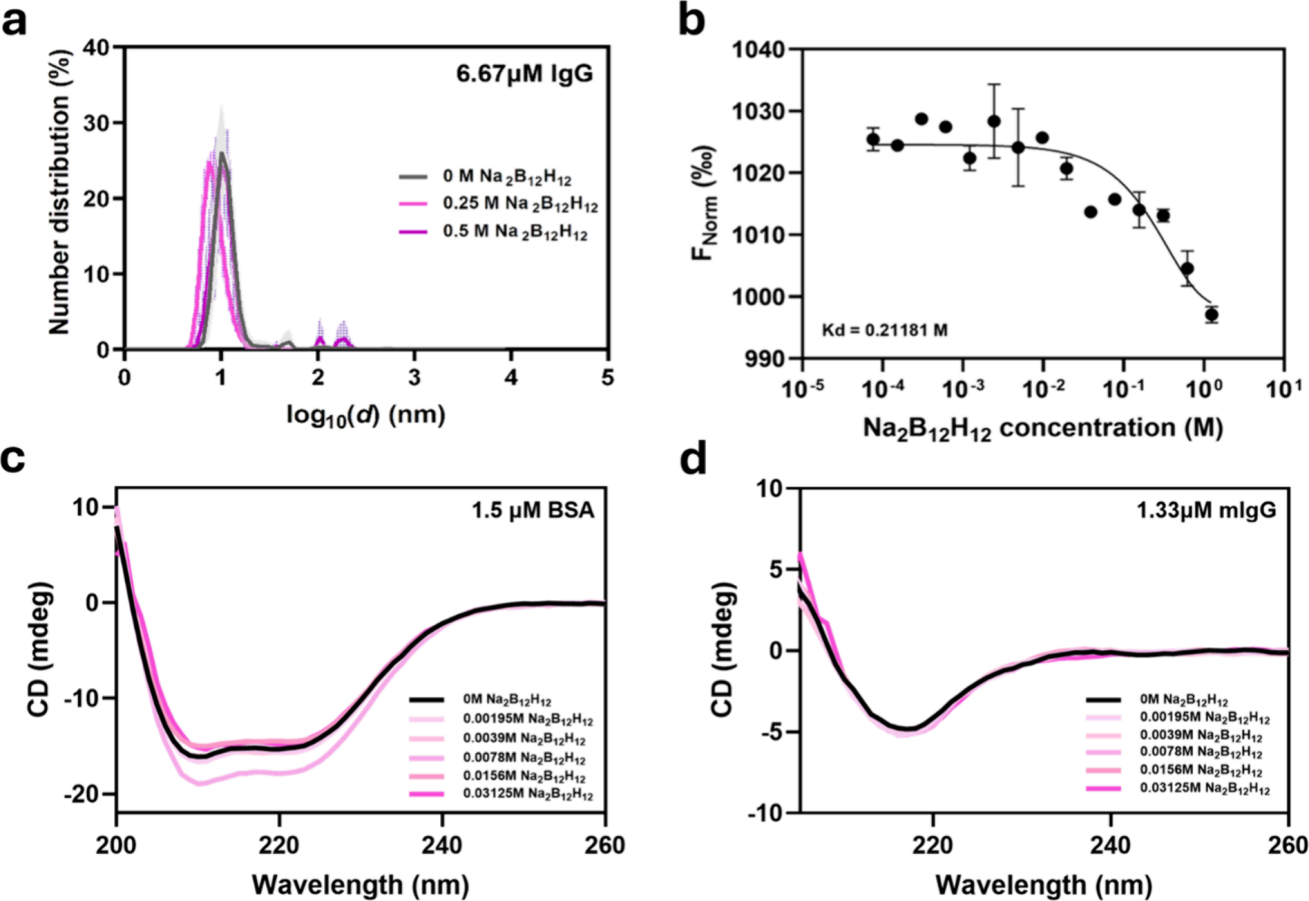
The weakly coordinating superchaotrope [B_12_H_12_]^2–^ does not cause protein denaturation at very high molar excesses. (a) Dynamic light scattering of 1 mg/mL IgG with varying [B_12_H_12_]^2–^ concentrations at 37 °C; the graph shows the percentage distribution of particles at various measured sizes. (b) Microscale thermophoretic changes of IgG with varying concentrations of [B_12_H_12_]^2–^. (c,d) Circular dichroism spectra of 0.1 mg/mL BSA and IgG at varying [B_12_H_12_]^2–^ concentrations. *N* = 3 experimental replicates at each [B_12_H_12_]^2–^ concentration step.

It is possible that [B_12_H_12_]^2–^ denatures protein but prohibits their aggregation, which would evade denaturation detection by DLS. To test this complementary hypothesis, we tested the thermophoretic behavior of fluorescent-labeled IgGs at various concentrations of [B_12_H_12_]^2–^ using microscale thermophoresis (MST). MST probes fluorescently labeled macromolecular diffusion along a local thermal gradient, which is affected by the hydrodynamic radii of the fluorescent protein species.^33^ Its unique assay design enables a highly reproducible thermophoresis under varying buffer conditions. Unfolded proteins will have increased hydrodynamic radii and, hence, an altered thermophoresis response. Figure 2b shows the MST titration curve of labeled IgG at varying molar concentrations of [B_12_H_12_]^2–^. The thermophoretic behavior remains largely constant until hundreds of millimolar [B_12_H_12_]^2–^ are present, corroborating our DLS results that [B_12_H_12_]^2–^ does not induce protein denaturation and supporting [B_12_H_12_]^2–^’s weakly coordinating nature (*K*_d_ = 0.212 M). We note that MSTs do show changes in thermophoretic behaviors at 0.1 M concentration ranges, in contrast to our DLS findings. This can be due to one assay being more sensitive than the other or some other [B_12_H_12_]^2–^-affected physical properties of the system (such as viscosity and specific heat capacity) indirectly influencing the results of one assay more significantly.

Having confirmed that [B_12_H_12_]^2–^ does not promote protein denaturative aggregation and global unfolding, we next tested whether [B_12_H_12_]^2–^ would affect secondary structures in a protein. This can be measured using circular dichroism, which reflects changes in the chiral arrangement of the peptide bond in the far-UV region (180–260 nm) and UV-fluorescent protein residues such as aromatic, disulfide, and tryptophan residues in the near-UV region (260–320 nm).^34,35^ We measured circular dichroism (CD) spectra with increasing [B_12_H_12_]^2–^ concentrations for IgG and BSA. Figure 2c shows that CD spectra remained invariant up to 0.03125 M [B_12_H_12_]^2–^, representing >20,000-fold molar excess for both proteins. Higher concentrations were tested but proved unreliable due to intense UV light absorption by [B_12_H_12_]^2–^ at 160–200 nm (Supplementary Figure 2). This is in keeping with others’ observation that boron clusters do not affect secondary structural folds and retain their biological activity.^30,36^

Collectively, DLS, MST, and CD experiments confirm that [B_12_H_12_]^2–^ does not denature proteins despite its potent disruption of water’s hydrogen bonding network. This is likely attributable to its weak coordinative properties, in keeping with existing literature that destabilizing agents directly interact with proteins and favor the unfolded state.

### [B_12_H_12_]^2–^ Nondenaturatively Suppresses Associative Protein–Protein Interactions

While [B_12_H_12_]^2–^ does not affect intramolecular interactions, we explored its effects on intermolecular protein–protein interactions. Conventional chaotropes and detergents are known to promote the solvation of macromolecules via different physical mechanisms. Still, they all inevitably involve direct interaction with the macromolecules and, hence, promotion of protein denaturation. It will therefore be interesting to see if a weakly coordinating superchaotrope like [B_12_H_12_]^2–^ can still promote protein solvation and hence suppresses associative macromolecular interactions generally.

A simple readout shown in Figure 3a using enzyme-linked immunosorbent assay (ELISA) revealed reduced binding of monoclonal anti-His-tag antibody to immobilized His-tagged protein antigens. In this experiment, 62.5 mM [B_12_H_12_]^2–^ was added to anti-His-tag antibodies when they were allowed to bind to the assay wells, followed by ample washing and signal generation to detect the actual amount of antibodies bound to the antigen-coated wells. We repeated the experiment with progressively higher [B_12_H_12_]^2–^ concentrations; the results are shown in Figure 3b, where decreasing ELISA signals were observed with increasing [B_12_H_12_]^2–^ concentrations, suggestive of increasingly inhibited antibody–antigen interactions.

**Figure 3:**
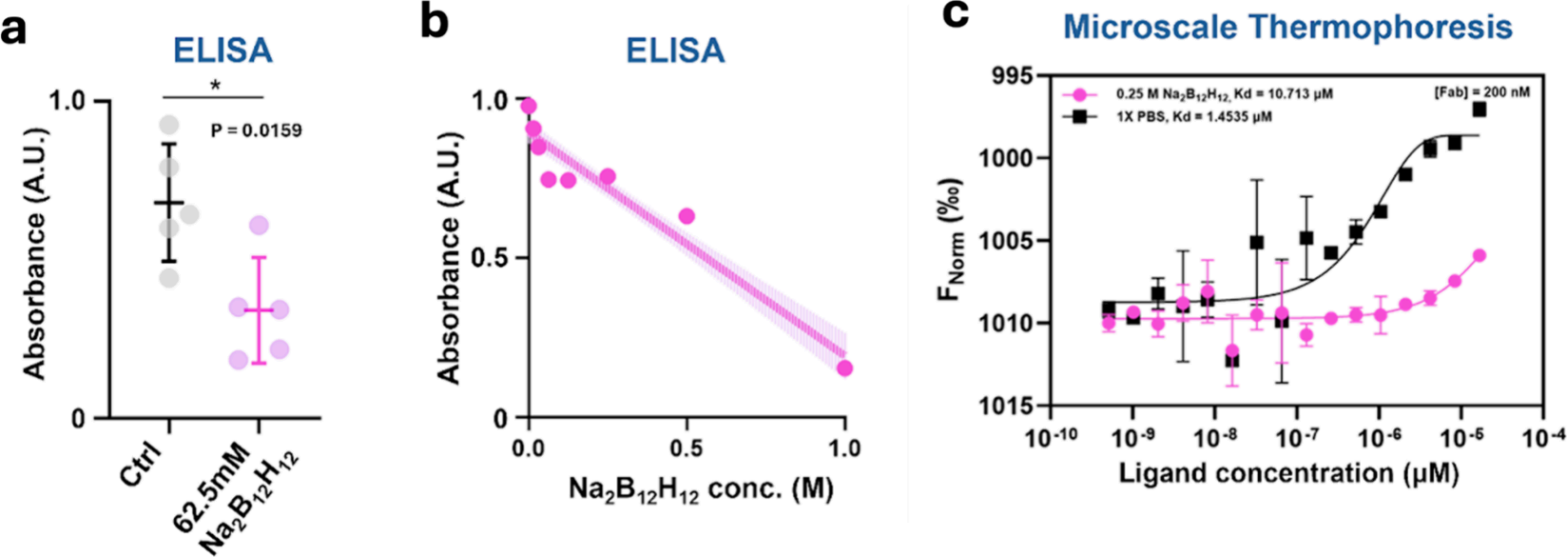
[B_12_H_12_]^2–^ broadly inhibits protein multimerization. (a) Comparing absorbance signal from the binding of mouse monoclonal anti-His_6_ IgG1 to immobilized His_6_-tagged protein in the absence or presence of 0.0625 M [B_12_H_12_]^2–^ in an ELISA. (b) Absorbance signal due to anti-His_6_ IgG1 binding to immobilized His_6_-tagged protein under varying [B_12_H_12_]^2–^ concentrations in an ELISA. Data points as pink dots, the linear regression curve as a pink line, and the line fitting 95% confidence interval as a pink shade. (c) Microscale thermophoresis monitoring of 200 nM polyclonal AlexaFluor 594-labeled polyclonal antirabbit IgG antibody Fab fragments binding its antigen rabbit IgG at varying concentrations, in the presence or absence of 0.25 M [B_12_H_12_]^2–^. The dissociation constant (*K*_d_) was obtained by fitting a sigmoidal curve. Error bars = s.d.; *N* = 2. The graph was inverted on the *y*-axis for a more consistent display of curve shifts with other panels due to reduced antigen binding.

We next utilized MST to obtain more quantitative data on the inhibition of protein–protein interactions by [B_12_H_12_]^2–^. As discussed, MST allows in-solution monitoring of protein–protein interaction affinity changes at dilute protein concentrations and arbitrary tuning of the [B_12_H_12_]^2–^ concentrations. This avoids interfacial effects on protein–protein interactions and the possibility that [B_12_H_12_]^2–^ might elute the adsorbed antigens from commercial ELISA plates. Figure 3c shows the MST results using fluorescently labeled antirabbit polyclonal antibody Fab fragments binding to rabbit IgGs. Strikingly, 0.25 M [B_12_H_12_]^2–^ reduced protein binding 7.37-fold compared to PBS control alone, as determined from thermophoretic behavior changes upon IgG titration (Figure 3c). Notably, the polyclonal nature of the antibodies suggests that these effects on protein–protein interactions are broadly applicable, in keeping with [B_12_H_12_]^2–^ as a cosolute species that exerts its effect via solvation modulation.

### Thermodynamic and Kinetic Effects of [B_12_H_12_]^2–^ on Protein Multimerization

Given the generality of protein–protein-associative interaction inhibition by the simple addition of the Na_2_B_12_H_12_ salt, we performed further experiments using other intermolecular binding models, where larger quantities of proteins are available commercially for thermodynamic and kinetic studies. We examined trypsin-ovomucoid inhibitor interactions in the presence of [B_12_H_12_]^2–^ using isothermal titration calorimetry (ITC). Consistent with previous findings, increasing [B_12_H_12_]^2–^ concentrations decreased the observed binding affinities. Figure 4a shows the fitted ITC curves with the original tracings provided in Supplementary Figure 3. Compared to the no-additive control, 0.25 M [B_12_H_12_]^2–^ reduced trypsin-ovomucoid affinity 5.24-fold. Such an effect was not seen with the same concentration of NaCl added.

**Figure 4:**
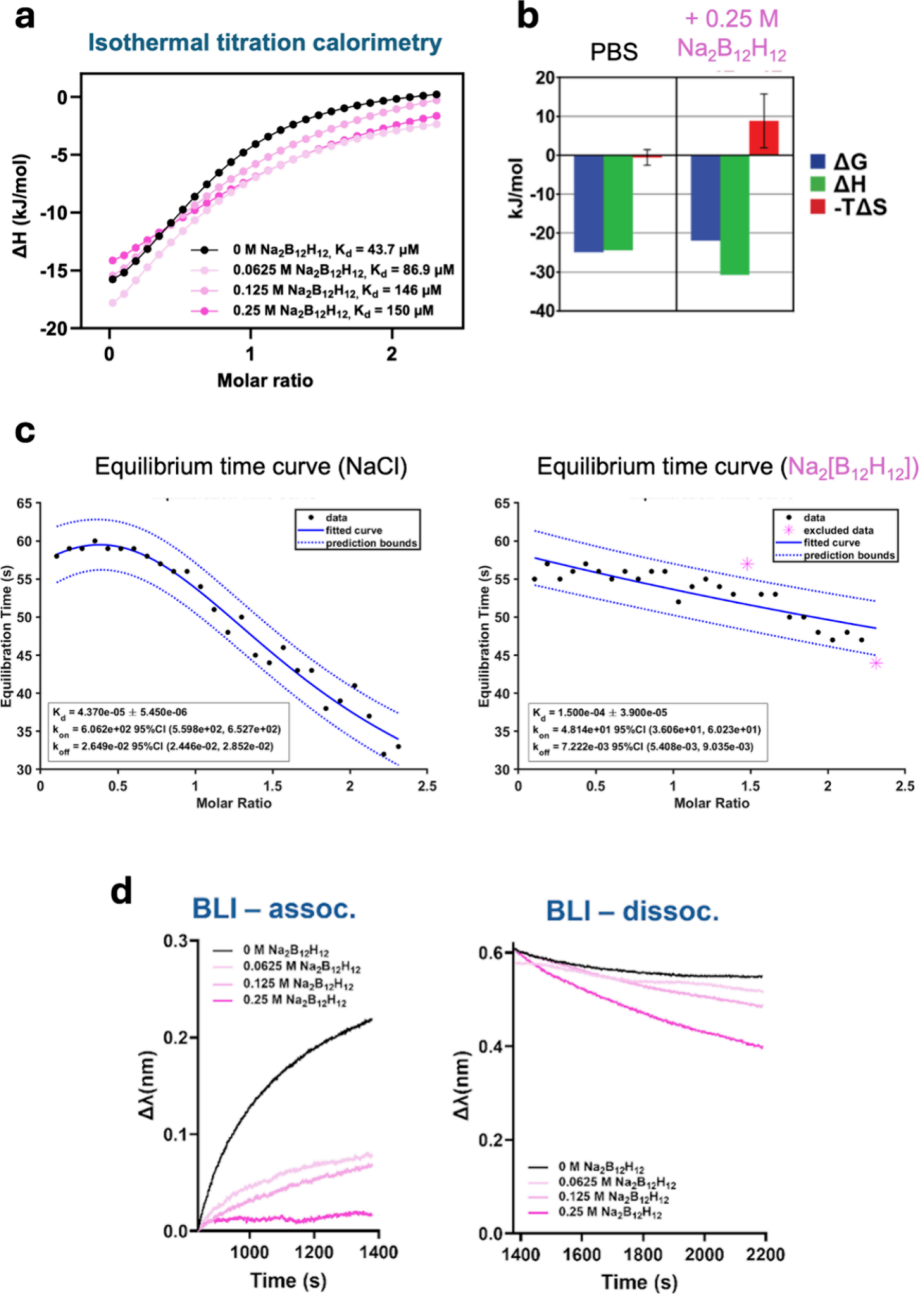
Thermodynamics and kinetics of protein–protein-associative interaction in the presence of [B_12_H_12_]^2‑^. (a) Isothermal titration calorimetry monitoring of the binding reaction between 1.8 mM trypsin and 0.15 mM ovomucoid at various concentrations of [B_12_H_12_]^2–^. (b) Thermodynamic analysis of ITC results in panel (a) for the 0 M Na_2_B_12_H_12_ group (PBS only) versus 0.25 M Na_2_B_12_H_12_ (added into addition to PBS) group. (c) Kinetic ITC (kinITC) analyses of trypsin and ovomucoid interaction in the presence of 0.25 M NaCl (left) and 0.25 M [B_12_H_12_]^2–^ (right). (d) Left: Biolayer interferometry monitoring of CALB1 binding to mouse monoclonal anti-CALB1 IgG1 immobilized on the probe at various concentrations of [B_12_H_12_]^2–^. Right: Biolayer interferometry monitoring of CALB1 dissociation from immobilized anti-CALB1 IgG1 at various concentrations of [B_12_H_12_]^2–^.

We compared the thermodynamics of trypsin-ovomucoid binding in 0.25 M Na_2_[B_12_H_12_] versus 0.25 M NaCl control. As shown in Figure 4b, [B_12_H_12_]^2–^ shifted the −*T*Δ*S* term toward positive (unfavorable) values with some compensatory increase in enthalpy. This overall loss of entropic gain in protein–protein binding is characteristic of chaotropic effects on macromolecular binding. This data should nonetheless be interpreted with caution due to the high concentration of protein required for clear ITC signals, and the system appears to be quite sensitive to the overall Na^+^ concentrations, as shown in Supplementary Figure 3. Kinetic data extracted from ITC experiments by kinetic ITC (kinITC) analysis,^37,38^ with the corresponding equilibrium time curves displayed in Figure 4c, indicate [B_12_H_12_]^2–^ reduced both association and dissociation rates between trypsin and ovomucoid, where a greater reduction in association rates is responsible for the overall decreased binding affinity between trypsin and ovomucoid.

Additional protein interaction kinetics were directly measured using biolayer interferometry (BLI).^39,40^ Protein binding is reflected by changes in refractive indices near the biosensor surface by using Fabry–Perot interferometry. We chose CALB1 and its binding partner, mouse anti-CALB1 monoclonal antibody, as the binding reaction model, where the latter was immobilized on BLI probes. Background subtraction was necessary as [B_12_H_12_]^2–^ substantially and reversibly shifted BLI curves (Supplementary Figure 4), likely due to its high UV absorption and changes in refractive index of the buffer solution rather than binding to protein-coated probes. Although this leads to uncertainties in curve fitting for the precise determination of binding rate constants, qualitative information can still be deciphered after background subtraction. The background-subtracted tracings are displayed in Figure 4d, which demonstrated decreasing binding affinity with increasing [B_12_H_12_]^2–^ concentrations. The curves reveal that [B_12_H_12_]^2–^ primarily decreased association rates (*k*_on_) while modestly increasing dissociation rates (*k*_off_). Comparison between 0.25 M Na_2_[B_12_H_12_] and 0.25 M NaCl (Supplementary Figure 5) confirmed that reduced association kinetics were attributable to [B_12_H_12_]^2–^ ions. These experiments establish that [B_12_H_12_]^2–^ primarily reduces protein–protein-associative interaction affinities by reducing association kinetics, with a more variable effect on dissociation kinetics.

### Enhancement of Colloidal Stability by Weakly Coordinating Superchaotrope

Our observation that [B_12_H_12_]^2–^ broadly inhibits specific protein–protein-associative interactions while weakly associating with proteins suggests that it may also inhibit nonspecific protein aggregation. As protein denaturation involves the formation of metastable unfolded intermediates, which aggregate over time, the noninteractive stabilization of protein monomeric state by [B_12_H_12_]^2–^ should enhance the stability of natively folded proteins in aqueous colloidal solutions with respect to both denaturation and aggregation.

Precise physical methods are generally unavailable for quantitatively measuring aggregative processes under diverse conditions. We tested our idea with various protein aggregation phenomena with a clear visual readout. We first used gelatin as a simple test. Gelatin consists of digested collagens,^41^ the aqueous solution that will solidify when cooled beyond its gelation temperature. This is due to the formation of a heterogeneous mixture of triple helices and amorphous aggregation of the interhelix regions, which involves protein desolvation and association interaction. We found that adding [B_12_H_12_]^2–^ to 2% gelatin prevented gelation when cooled to 4 °C (Figure 5a).

**Figure 5:**
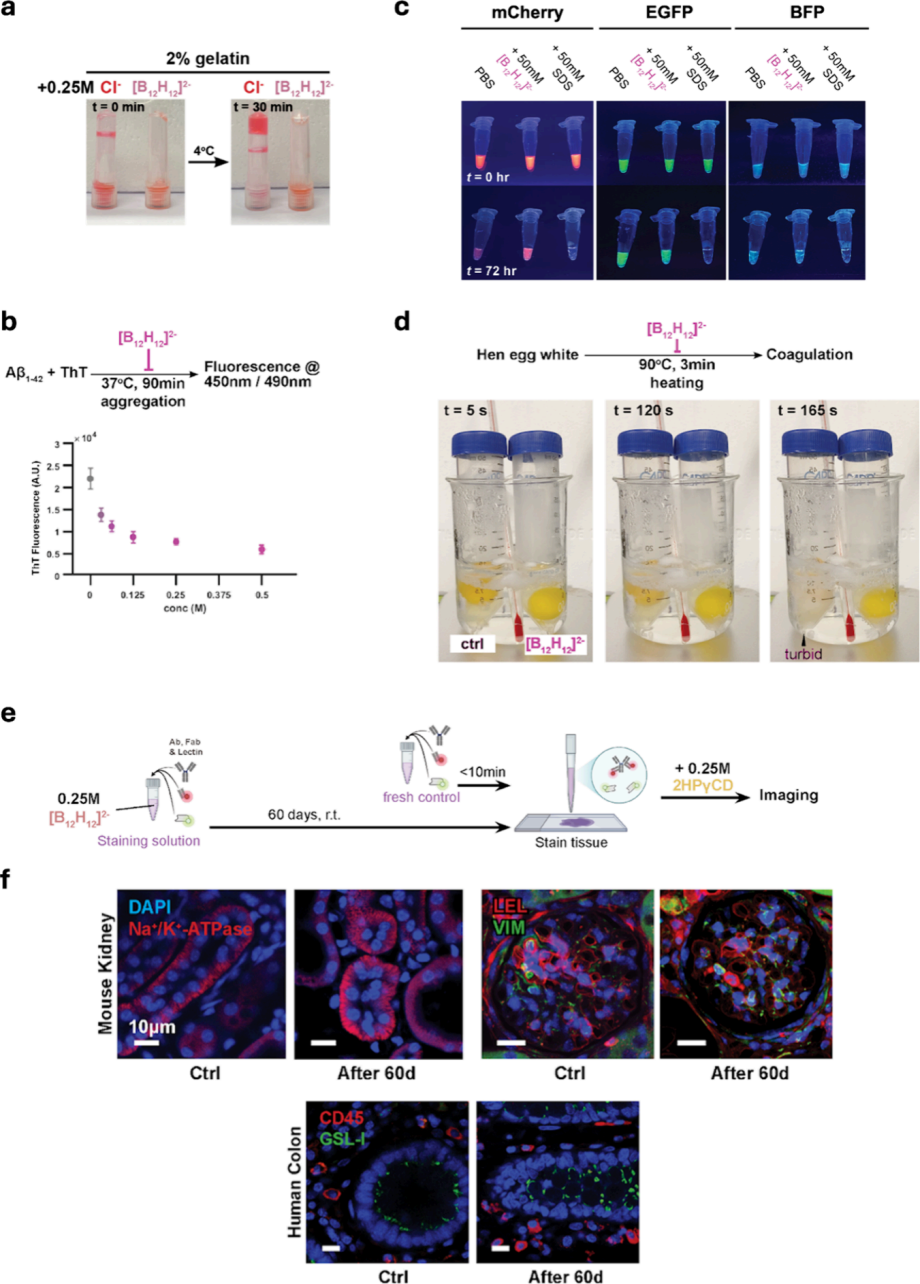
[B_12_H_12_]^2‑^preserves metastable colloids. (a) [B_12_H_12_]^2–^ inhibits the gelation of 2% gelatin compared to the same concentration of chloride ions. (b) Aggregation of Aβ_1–42_ peptide after 90 min of incubation under varying concentrations of [B_12_H_12_]^2–^, as measured by thioflavin T fluorescence. Higher fluorescence indicates more Aβ_1–42_ aggregates formed. (c) Fluorescent proteins retain their fluorescence properties after incubating at 55 °C in the dark for 72 h in the presence of [B_12_H_12_]^2–^. (d) Delayed heat-induced coagulative changes due to the addition of [B_12_H_12_]^2–^ to a final concentration of 0.1 M in 1:1 saline-diluted hen egg white. At *t* = 165 s, the control hen egg turned opaque during cooking, while the [B_12_H_12_]^2–^-treated egg white remained translucent. (e,f) Fluorescently labeled antibodies and lectins are stable in 0.25 M [B_12_H_12_]^2–^ after storing at room temperature for 60 days. The upper schematic shows the experimental design where a panel of primary antibody (Ab), fluorophore-labeled secondary antibody Fab fragment (Fab), and fluorophore-labeled lectins (Lectin) were added to 1× PBS with 0.25 M [B_12_H_12_]^2–^ and stored at room temperature (r.t.) for 60 days in the dark. For control, the same solution was prepared freshly and used immediately. The staining solutions were then applied to tissues, after which it was washed with 1× PBS with 0.25 M 2HPγCD, before proceeding to imaging. The lower images show the staining results of the various applied markers in mouse kidney and human colon tissues. LEL: *Lycopersicon esculentum* lectin, GSL-I: *Griffonia simplicifolia* lectin I.

We next tested beta-amyloid-(1–42) peptide (Aβ_1–42_) aggregation in the presence of [B_12_H_12_]^2–^. Aβ_1–42_ is widely known for its role in the formation of amyloid plaques in Alzheimer’s disease and senile dementia. Its fibrillation process is known to be hydrophobically driven^42^ and the prevention of which is challenging with traditional structure-guided design^43^ even when the protein structures were well-resolved. To our delight, [B_12_H_12_]^2–^ suppressed Aβ_1–42_ aggregation in a dose-dependent manner, as demonstrated in Figure 5b using the thioflavin T (ThT) binding assay, where a fluorescence signal is generated when ThT binds to aggregated amyloid fibrils.^44^ Supplementary Figure 6 confirms [B_12_H_12_]^2–^ alone does not affect ThT binding to Aβ_1–42_ fibrils nor its fluorescence response, yet suppresses amyloid fibril formation.

Apart from testing protein aggregation and gelation phenomena under native conditions, we further tested protein aggregation under denaturating conditions at high temperatures. We tested 50 mM [B_12_H_12_]^2–^ with recombinant fluorescent proteins incubated at 55 °C for up to 72 h, using UV-illuminated fluorescence to probe structural preservation (Figure 5c), as a native β barrel fold is essential for their fluorescence.^45^ We included sodium dodecyl sulfate (SDS, 50 mM) as a surfactant-based control. Remarkably, [B_12_H_12_]^2–^ preserved fluorescence better than did SDS and better preserved fluorescence than did PBS for mCherry. Apart from fluorescent proteins, we further tested protein thermal denaturation using hen egg white by heating at 95 °C, which leads to coagulation and opacification in a familiar cooking process. Figure 5d and Supplementary Video 1 show that the addition of [B_12_H_12_]^2–^ delayed protein coagulative aggregation compared with the control having the same molar concentrations of Cl^–^. Although inevitably proteins proceed to denaturation under continuous thermal stress, it is clear that the superchaotrope [B_12_H_12_]^2–^ does not hasten protein denaturation and in some cases even partly stabilizes them even under sustained heating.

The maintained colloidal and protein stability that emerges from the prevention of both specific and nonspecific intermolecular interactions suggests [B_12_H_12_]^2–^ can serve as a preservative for multicomponent protein mixtures under fluctuating temperature conditions upon long-term storage. To test this hypothesis, we prepared fluorescent histochemical staining solutions containing primary antibodies, fluorophore-labeled secondary antibody Fab fragments, and fluorophore-labeled lectins in PBS with 0.25 M [B_12_H_12_]^2–^, storing them at ambient temperatures (15–25 °C) for 60 days, illustrated in Figure 5d. After [B_12_H_12_]^2–^ was removed by ultracentrifugal filtration, the recovered solutions were applied to mouse kidney and human colon tissues. We observed no fluorescent precipitates at the bottom of the ultracentrifugal filter. As shown in Figure 5e, despite containing high concentrations of proteins and hydrophobic solutes without refrigeration, the aged solutions produced high-quality histochemistry results comparable to those of freshly prepared controls. Due to their unstable multiple domains and aggregation propensity even in the folded state, IgGs, especially fluorescently conjugated ones and Fab fragments,^46^ are notoriously known to be unstable even when stored in aqueous solution at 4 °C. Although multiple chemical and formulation engineering approaches have been developed,^47–54^ adding [B_12_H_12_]^2–^ as a nonhazardous and chemically inert additive is a simple, affordable, and universal way to preserve protein formulation stability. Furthermore, its usage will be advantageous as solid-supported supramolecular hosts^55,56^ (e.g., γ-cyclodextrins) can be used to remove [B_12_H_12_]^2–^ on-demand selectively, without requiring the use of specialized conditions or equipment for liquid formulation reconstitution.

## Discussion

Our findings reveal a paradoxical behavior of the superchaotropic [B_12_H_12_]^2–^ ion that challenges conventional understanding of the Hofmeister series effects on protein stability.^57,58^ Despite its suggested potent chaotropicity based on its low charge density-to-radius ratio^29,55,59,60^ and the thermodynamics of its reaction with supramolecular hosts in aqueous conditions,^28,56^ which would traditionally suggest protein destabilization, [B_12_H_12_]^2–^ actually preserves protein conformational integrity while simultaneously inhibiting protein–protein-associative interactions. This unique dual functionality is distinct from the known arsenal of protein-stabilizing and protein-solubilizing agents, where favoring protein stability also seems to promote protein–protein-associative interaction. The segregated properties of being weakly coordinating and superchaotropic for [B_12_H_12_]^2–^ lead to the favoring of natively folded but monomeric protein states. This effect is applicable to a wide range of proteins with diverse properties (summarized in Supplementary Table 1) and seems robust across a wide range of conditions tested in this study, even under extreme tests, such as the boiling of hen egg white protein. It is likely that protection against aggregation and irreversible denaturation by [B_12_H_12_]^2–^ may result from its ability to prevent the early stages of protein misfolding cascades and aggregate nucleation. The importance of weak coordination is perhaps pivotal, as other radially symmetric or asymmetric boron clusters are also nondenaturative even though they were shown to interact strongly with proteins,^29,36,56,61^ in keeping with statistical mechanical theories (Supplementary Notes). Overall, the ability of [B_12_H_12_]^2–^ to globally reduce protein–protein-associative interactions has significant implications for protein storage and stability, as illustrated by the remarkable preservation of complex antibody mixtures at ambient temperatures.

Our preliminary mechanistic investigation suggests that [B_12_H_12_]^2–^ appears to increase the entropic cost of protein–protein association without destabilizing individual protein folding, as evidenced by our thermodynamic analyses. Future investigations with other protein–protein interaction systems with more robust ITC signals will help clarify the thermodynamic effects of [B_12_H_12_]^2–^ on protein–protein interactions. Combined with our kinetic data that [B_12_H_12_]^2–^ primarily affects association rates rather than dissociation processes, this suggests [B_12_H_12_]^2–^ creates an energetic barrier to initial protein–protein contact formation, possibly affecting the desolvation dynamics at protein surfaces.^62^ Once bound, protein–protein complexes seem to remain relatively stable. However, much further work remains to be done to explore the mechanism. A key question is whether [B_12_H_12_]^2–^ is preferentially excluded around macromolecules (i.e., [B_12_H_12_]^2–^ is preferentially dissolved in bulk water solvent), even when it does not directly interact with protein residues, where recently developed nuclear magnetic resonance techniques will be helpful.^62^ If [B_12_H_12_]^2–^ is preferentially excluded from protein surface, the stabilizing mechanism can be expected to be similar to that of other osmolytes and molecular crowding agents; however, it would be more elusive on how [B_12_H_12_]^2–^ inhibits protein–protein-associative interactions, as the release of surface bound water to a bulk solution containing a chaotrope should lead to substantial entropic gains. If [B_12_H_12_]^2–^ preferentially accumulates around proteins, which has been demonstrated for nonionic surfaces and solutes,^63,64^ it may form an electrostatic barrier that leads to macromolecular-boron cluster assembly repulsion, implying a novel protein stabilization mechanism by surface dehydration that somewhat resembles hydrotropy.^65^ Colloidal stabilization of detergent bilayers by superchaotropes adsorbed on hydrophobic reagents has been observed due to electrostatic repulsion.^66,67^ Detailed postulations linking statistical mechanical theorization efforts are provided in the Supplementary Note, although we stress that the hypotheses are only speculated based on the best available evidence.

The practical applications of this discovery extend beyond basic protein science. Stabilizing proteins while preventing their aggregation and interaction could benefit biopharmaceutical formulation, enzyme stabilization in industrial processes, and the development of novel diagnostic reagents with extended shelf life at ambient conditions.^68^ This gives [B_12_H_12_]^2–^ immediate application to real-world problems. In addition, in preventing the formation of amyloid fibrils, [B_12_H_12_]^2–^ opens up a novel motif that can be covalently linked to small molecules for a spatially confined weakly coordinating superchaotropic prevention or dissociation of fibrils, thanks to the mature derivatization chemistry available.^69^ Substantial research has demonstrated the safety of [B_12_H_12_]^2–^ and its derivatives in *in vivo* settings^70^ during exploring boron neutron capture therapy (BNCT) agents.^71,72^ Our discovery can augment these works for the localized modulation of macromolecular interactions.^73–108^

Future research should explore structure–activity relationships of similar superchaotropic ions, investigate concentration-dependent effects in diverse protein systems, and elucidate the molecular details of the solvation dynamics in these unusual electrolytes by including more protein species. Additionally, combining [B_12_H_12_]^2–^ with traditional stabilizing agents might yield synergistic effects for extreme protein preservation needs.

## Conclusions

In conclusion, the superchaotropic [B_12_H_12_]^2–^ ion represents a novel tool for protein stabilization that works through a mechanism distinct from traditional kosmotropes and chaotropes. By preserving protein conformations while inhibiting protein–protein-associative interactions, [B_12_H_12_]^2–^ offers unique capabilities for controlling protein behavior in solutions, with promising applications across biotechnology, pharmaceutical sciences, and research methodologies.

## Supplementary Material





## Data Availability

Additional experimental data used in this study will be available upon reasonable request to the corresponding author.

## Notes

Supplementary theoretical postulations, DLS results, CD results, ITC results, BLI results, ThT fluorescence results, summarized protein information table (PDF)
Biomacromolecule protein stabilization illustrated by boiling hen egg white. (MP4)
