# Supplementary material for: Superchaotropic Stabilization of Monomeric Protein States
Source: Biomacromolecules. 2025 Sep 1;27(2):1138–49. doi: 10.1021/acs.biomac.5c00944 (PMC12892320; doi:10.1021/acs.biomac.5c00944)
Supplement: Supplementary file 1 [file bm5c00944_si_001.pdf]

Supporting Information

Superchaotropic stabilisation of monomeric protein states

Ben Tin Yan Wong[1-3], Lichun Zhang[1-3†], Thomas Chun Yip Wong[2,3†], Chun Ngo Yau[3], Adrian Jun Chu[5], Tsz Fung Tsang[5], Joshua Jing Xi Li[6], Xiao Yang[5], Hei Ming Lai[1-4*]

Affiliations

[1]Department of Chemical Pathology, Faculty of Medicine, The Chinese University of Hong Kong, Shatin, Hong Kong SAR, China.

[2]Illumos Limited, Hong Kong Science and Technology Park, Shatin, Hong Kong SAR, China.

[3]Li Ka Shing Institute of Health Sciences, Faculty of Medicine, The Chinese University of Hong Kong, Shatin, Hong Kong SAR, China.

[4]Department of Psychiatry, Faculty of Medicine, The Chinese University of Hong Kong, Shatin, Hong Kong SAR, China.

[5]Department of Microbiology, Faculty of Medicine, The Chinese University of Hong Kong, Shatin, Hong Kong SAR, China.

[6]Department of Pathology, School of Clinical Medicine, The University of Hong Kong, Queen Mary Hospital, Hong Kong SAR, China.

[†]Equal contributions

[*]Corresponding authors. Email: hmlai@cuhk.edu.hk

Supplementary Information

Supplementary Notes
Supplementary Figures 1 - 6
Supplementary Table 1
Supplementary Video captions

Supplementary Notes

Supplementary Note 1. Hypotheses on the mechanism of action of $[B_{12}H_{12}]^{2-}$ as a weakly coordinating superchaotrope.

The molecular mechanism of how $[B_{12}H_{12}]^{2-}$ leads to a broad and general decrease in specific and non-specific macromolecular interactions remains incompletely understood. Existing literature primarily focuses on co-solutes' effects on protein folding stability(17), with fewer systematic studies investigating their influence on protein-protein interactions. Therefore, concluding a generalised mechanism of $[B_{12}H_{12}]^{2-}$ of action is premature as this aspect of data is severely lacking. Here, we outline a theoretical framework, using rigorous statistical mechanics—particularly Kirkwood-Buff (KB) theory(12)—to interpret observable phenomena and clarify the proposed role of $[B_{12}H_{12}]^{2-}$.

Protein in a water-co-solute system

When a protein is placed in a mixed solvent of water and another co-solute, the resulting thermodynamic behaviour can be significantly influenced by where the co-solute and water prefer. They can be

- Preferentially excluded from the neighbourhood of a protein, equivalent to being more solvated in the bulk solvent than being near the protein surface, or
- Preferentially accumulated around the protein. This does not necessarily mean they bind to the proteins, but it can also mean solvation in the bulk solvent is relatively unfavoured.

These two scenarios can, in turn, stabilise or destabilise a protein's structure or assembly(17).

Kirkwood-Buff theory

Consider a solution containing water, protein, and a co-solute indexed as species 1, 2, and 3, respectively. Let n_i denote the molar concentration of species i in the bulk, and μ_i its chemical potential. For the case of $n_2 \rightarrow 0$, we have the pair correlation function $g_{ij}(r)$ between species i and j, describing how the local density of species j differs from the bulk density, as a function of distance r from a reference molecule of species i. From $g_{ij}(r)$, the Kirkwood-Buff theory defines an excess number N_{ij}:

$$N_{ij} = n_j N_A \int [g_{ij}(r) - 1] \, d\mathbf{r}$$

where N_A is Avogadro's number, n_j is the bulk concentration of species j, and the integral is over all space surrounding a reference molecule i. In this context of a dilute protein solution, N_{23} thus

measures how many co-solute molecules, in excess or deficit compared to a random distribution, surround one protein molecule. For example, $N_{23} < 0$ implies that, net, co-solute is excluded, while $N_{23} > 0$ implies preferential accumulation.

Technically, to remove the protein's core volume, a corrected "shell" or "net" excess solvation number $N'_{2j} = N'_{2j} + n_j V_E$, where V_E is the effective excluded (or inaccessible) volume. This is important for relating theory to measured volumes(14).

Preferential interaction parameters

If we track how the protein's chemical potential μ_2 changes as the water's chemical potential μ_1 or the co-solute's chemical potential μ_3 shifts, one can define a preferential interaction parameter (Γ_{21} or n_{21} in different notations in the literature) as

$$\Gamma_{21} = -\left(\frac{\partial \mu_2}{\partial \mu_1}\right)_{T,P,\mu_3} = N_{21} + \frac{n_1}{n_3} N_{23}.$$

If $n_{21} > 0$, the protein is preferentially hydrated, while $n_{21} < 0$ means the co-solute is preferentially accumulated at the protein surface(14, 73).

Experimental measurement of preferential interaction parameters

The power of KB theory lies in its ability to link microscopic quantities (e.g., excess numbers and preferential interaction parameters) to experimentally measurable macroscopic quantities, such as densities, partial molar volumes and compressibilities. Notably, it can be shown that the partial molar volume of the protein $\overline{V}_2$ is related to N_{21} and N_{23} by

$$\overline{V}_2 = \overline{V}_1 N_{21} + \overline{V}_3 N_{23} + RT\kappa_T$$

where R is the universal gas constant, T is the absolute temperature, and κ_T is the isothermal compressibility of the solution(74).

A note on chaotropes, kosmotropes, and hydrotropes terminology

Historically, co-solutes have been classified as kosmotropes (structure-promoting, osmolytes), chaotropes (structure-disrupting), or hydrotropes (solubilisers). However, these descriptors are context-dependent and can oversimplify complex behaviours.

While not absolute, experimentally, kosmotropes and osmolytes (e.g., sugars, polyols) are typically excluded from protein surfaces ($N_{23} < 0$), stabilising folded states via preferential hydration. Chaotropes (e.g., urea, thiocyanate) were often found to preferentially accumulate at

protein surfaces ($N_{23} > 0$) and destabilize proteins via direct interactions with residues, leading to enthalpic destabilization of protein folding(75). Meanwhile, hydrotropes promote protein solubility by local accumulation near hydrophobic regions without necessarily unfolding proteins(76–79).

Note that these categorization is not absolute, as some salts (e.g., ammonium sulfate) exhibit behavior dependent on protein identity and conditions. Hence, below we explicitly discuss co-solutes with reference to their physical effects on protein stability and protein-protein interactions.

Protein-stabilizing co-solutes

Experimental evidence have suggested that preferential hydration is a key mechanism behind protein-stabilising co-solutes, that is, their presence leads to $\Gamma_{21} > 0$. We have the free energy of folding in the presence of co-solute, approximated based on partitioning models, as

$$\Delta G_{\text{fold}}^{\text{co-solute}} = \Delta G_{\text{fold}}^{\text{water}} + RT \ln(1 + \Gamma_{21})$$

where $\Delta G_{\text{fold}}^{\text{water}}$ is the folding free energy in pure water(13, 14).

Protein-destabilizing co-solutes

In contrast, co-solutes that destabilise proteins were experimentally found to accumulate near protein surfaces preferentially, i.e., $\Gamma_{21} < 0$. The free energy of folding is approximated as(13, 16, 17)

$$\Delta G_{\text{fold}}^{\text{co-solute}} = \Delta G_{\text{fold}}^{\text{water}} - RT \ln(1 - \Gamma_{21}).$$

Co-solutes and protein-protein interactions

For an interaction reaction between protein A and protein B in aqueous environment, we have the following reaction,

$$\text{A}\cdot n\ \text{H}_2\text{O} + \text{B}\cdot m\ \text{H}_2\text{O} \rightarrow [\text{A-B}]\cdot(n+m-i-j)\ \text{H}_2\text{O} + (i+j)\ \text{H}_2\text{O}$$

With the free energy change of association given by $\Delta G_{\text{assoc}} = -RT \ln K_a$, where K_a is the affinity constant of the reaction, and can be decomposed into components of direct protein-protein non-covalent interactions, desolvation due to removed protein-water interactions, and configurational changes. In an aqueous environment, desolvation is often a significant term, contributing roughly $\Delta G_{\text{desolv}} \approx -(i+j)\ \Delta\mu_{\text{H2O}}$, as these expelled water molecules move from a preferentially accumulated state to the bulk state with differing chemical potentials $\Delta\mu_{\text{H2O}}$. Considering the relatively slow diffusion of macromolecules compared to water, and the wide variations of the water molecules'

residence time on protein surfaces, computational simulations have demonstrated that desolvation can alter protein-protein interaction kinetics by a thousand-fold(80–82). Hence, there is an association-desolvation equivalence where the solubilisation of proteins can affect associative reactions.

In hydrotropy, certain co-solutes were found to prevent the aggregation and phase separation of proteins(76–79). This can be understood, in Kirkwood-Buff terms, as preferential solute-protein interactions that enhance the protein's overall hydration(65). In other words, the crowding of hydrotrope near the protein surface modifies its interfacial interaction with water locally, indirectly promoting solubilisation and preventing protein-protein aggregation. This has been confirmed experimentally(83, 84) and computationally(85, 86) with $N_{23} > 0$ for the case of protein solubilisation with urea, or a positive "hydrotrope-protein" preferential interaction parameter $\Gamma_{[23]1} > 0$, where we denote [23] as the statistical assembly of the protein 2 and the co-solute 3.

Correlation between protein denaturation and direct interaction

For protein-destabilising co-solutes such as thiocyanate, urea, and guanidinium, a significant destabilising effect can be attributed to the direct co-solute interaction with protein residues, which is enthalpically driven and independent of preferential interactions. Meanwhile, although macromolecular crowders stabilise proteins by being preferentially excluded from the protein surfaces, detailed thermodynamics investigations revealed they can also destabilise protein folding if they have strongly enthalpic directional interactions with the protein residues(19, 87–89). Hence, the key to non-denaturative hydrotropy - which preserves protein folding while suppresses protein-protein interactions, can be interpreted as avoiding direct interactions with proteins, while promoting hydrotropy by statistical preferential accumulation around proteins. Indeed, symmetric boron clusters $[B_{10}H_{10}]^{2-}$ and $[B_{12}H_{12}]^{2-}$ and their derivatives, and an asymmetric boron cluster derivative, which is also likely weakly coordinative, was found to strongly interact with, but not denature cytochrome *c* or cytotoxic peptides (36, 61).

Mathematically, consider urea, which directly interacts with proteins but also exhibits hydrotropy. Its dual role can be decomposed into

$$\Gamma_{21} = \Gamma_{21}^{\text{hydration}} + \Gamma_{21}^{\text{direct}}.$$

Here, $\Gamma_{21}^{\text{hydration}} > 0$ indicates indirect hydration effects consequential of the hydrotropy-induced accumulation of the co-solute (for $N_{23} > 0$), while $\Gamma_{21}^{\text{direct}} < 0$ indicates water displaced by the co-solute due to its direct interactions with protein residues, leading to unfolding.

Now, considering the net effect, if the indirect hydration term dominates, that is,

$$|\Gamma_{21}^{\mathrm{hydration}}| > |\Gamma_{21}^{\mathrm{direct}}|$$

We can have a co-solute that prevents protein-protein interactions without denaturation.

Kinetic inhibition of macromolecular binding and the preferential positioning of $[B_{12}H_{12}]^{2-}$

The observation that $[B_{12}H_{12}]^{2-}$ reduces protein interaction affinity by consistently reducing associative kinetics suggests further mechanisms in action. From the perspectives of collision theory, a successful interactive reaction depends on two molecules approaching each other in an appropriate orientation and momentum. The structuredness of water may provide a guided path for two macromolecules to approach each other with a constrained degree of freedom, and the disruption of which may lead to higher stochasticity in the gradient of chemical potential for the molecule to descend towards interaction(62, 90, 91).

As $[B_{12}H_{12}]^{2-}$ decreases the mean number of hydrogen bonds in water, one should expect a lower viscosity(92, 93) and hence higher collision rates and increased association kinetics with increased dissociation kinetics as the main cause of decreased affinity. The observed slowed associative kinetics by $[B_{12}H_{12}]^{2-}$ but with relatively little effect on the dissociative kinetics, suggest that the solvent structure is less permissive for collision to happen. As large, relatively rigid molecules, protein collisions are greatly influenced by the statistical structure of the solvent, which may serve to "guide" effective collisions between macromolecules(62, 90, 91). Conversely, the presence of the protein molecule as a dielectric body also perturbs the local solvent structure and may facilitate its associative processes.

From statistical mechanics, placing an electrostatically charged rigid molecule in a dielectric medium will lead to a reactive, directional electric field within the rigid cavity, termed the reactive field by Onsager(94), which has been further refined by Kirkwood and Fröhlich(95). By extension, one can therefore also expect the rigid electrostatic body of protein constrain the orientations accessible by dielectric molecules proximal to the protein, such as water molecules and its protein binding partners. This is quantified by Kirkwood's correlation factor g_K (not to be confused with the Kirkwood-Buff Integral, G_{ij}), which is defined as(96)

$$g_K = \frac{\langle \boldsymbol{\mu_e} \cdot \overline{\boldsymbol{\mu_e}} \rangle}{\mu^2} = 1 + \sum_{j \neq 0} \langle \cos \vartheta_{\alpha j} \rangle$$

where the protein located at the center of the system is denoted as molecule α, and the term $\boldsymbol{\mu_e}$ represents the total dipole moment of the system within the defined volume V. The term $\overline{\boldsymbol{\mu}_e}$ is the equilibrium-averaged total dipole moment, and μ is the magnitude of the dipole moment of an individual molecule. The quantity $\langle \cos \vartheta_{ij} \rangle$ represents the average cosine of the angle ϑ_{ij} between the dipole moments of molecules *i* and *j*, averaged over all orientations of the molecular

pairs of interest extending over a bounded volume around the protein α.

The perturbed water structure is then linked to the macroscopic dielectric constant by the Onsager-Kirkwood-Fröhlich equation, providing a means to study the water structure perturbation by dielectric spectroscopy(96):

$$\frac{(\varepsilon - \varepsilon_\infty)(2\varepsilon + \varepsilon_\infty)}{\varepsilon(\varepsilon_\infty + 2)^2} = \frac{4\pi\rho}{9k_BT} g_K \mu^2$$

Where ε is the static dielectric constant of the liquid, ε_∞ is the high-frequency dielectric constant of the liquid, ρ is the number density of molecules. If solvent-guided protein assembly is crucial for the associative kinetics of proteins in specific and non-specific interactions, then $[B_{12}H_{12}]^{2-}$ can potentially disrupt this process, even when it is not preferentially accumulated around proteins. Such an effect should be increasingly observable with increasing area of the interacting macromolecules. For example, the solvent-mediated long-range forces emerging from the solvent molecule dipoles can affect nanoparticle assembly beyond simple electrostatic explanations(97).

If, in contrast, $[B_{12}H_{12}]^{2-}$ preferentially accumulates around proteins, then the electrostatic repulsion between $[B_{12}H_{12}]^{2-}$ shielded proteins could provide a simple explanation for its broad anti-macromolecular assembly effects. This has been observed for polyoxometalates, which are also superchaotropic and provide a stabilizing force for detergent foams(66, 67). Preferential accumulation of $[B_{12}H_{12}]^{2-}$ ions around protein surfaces can be plausible if electrostatic interactions and protein hydrogen-hydride bonds prove to be more favourable than the water hydrogen-hydride bonds (98, 99). and positively charged protein residues are plausible at neutral pH, and there has been evidence for a strong dihydride bond between In addition, the local hydration shell would probably be pre-disrupted with a high initial entropy in the presence of $[B_{12}H_{12}]^{2-}$, leading to a statistical "dehydration" of the protein surface. Hence, as protein binds to each other and liberate the interfacial hydration shell, there will be fewer displaced water molecules, which also gain less entropy in migrating towards the bulk solvent, explaining our observed unfavourable entropy term in our trypsin-ovomucoid binding model. The preferential and likely highly dynamically association of $[B_{12}H_{12}]^{2-}$ with protein surfaces can be difficult to assess, however, recent groundbreaking developments in nuclear magnetic resonance (NMR) spectroscopy techniques may provide invaluable data and hence insights into how broad suppression of protein-protein interactions take place(84). Finally, in the most extreme mechanism, it has been shown that the theta-shaped detergent cobalt bis(7,8-dicarbollide) (*o*-COSAN) (100) where a "shield" is constructed around the protein that leads to its stabilization (101).

We reiterate that the above stated mechanisms are only speculative and derived based on the best available evidence. Nonetheless, $[B_{12}H_{12}]^{2-}$ can serve as a tool to dissect the role of solvent in macromolecular interaction, as it provides the first opportunity where protein denaturation as an

interference is removed, allowing examination of the solvent role in protein associative kinetics.

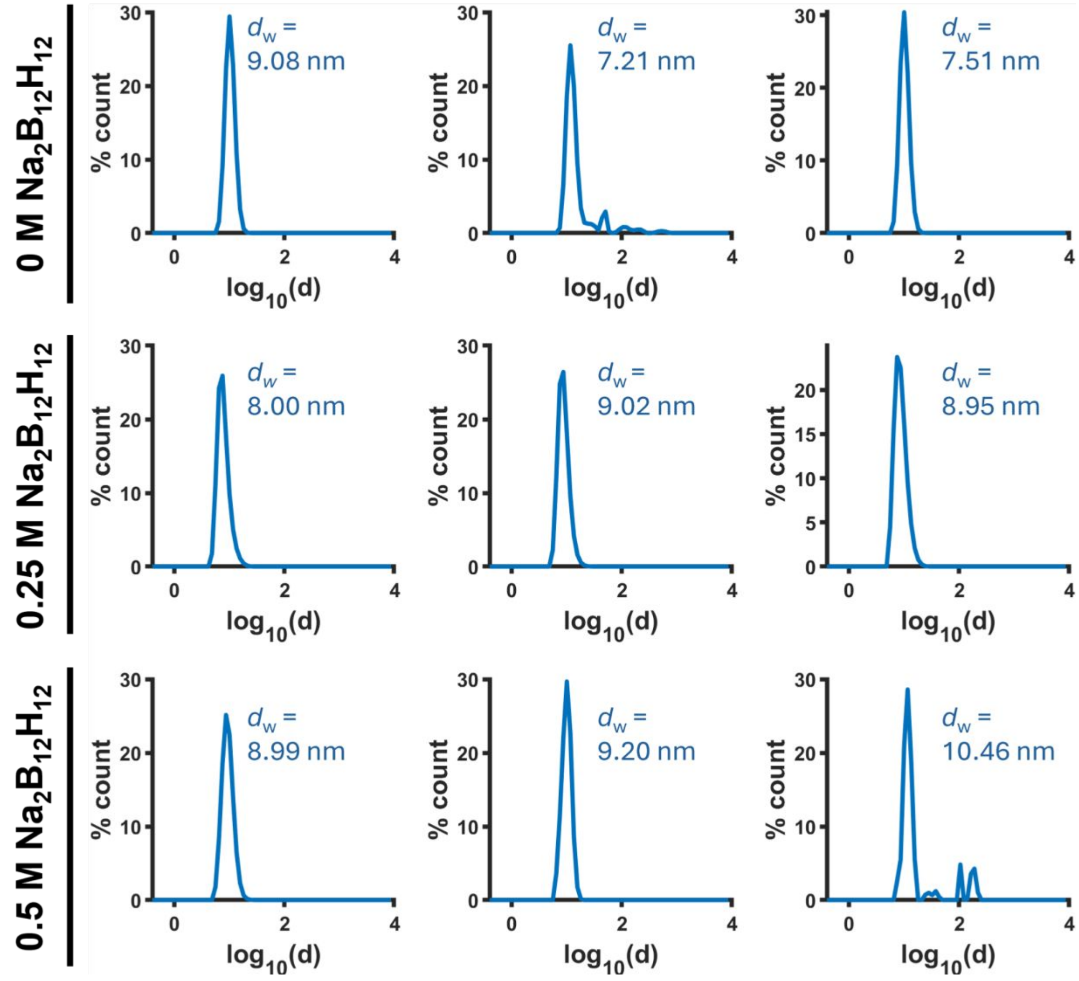

Supp Fig 1. **Individual dynamic light scattering experimental results**. Each of 3 replicates for each concentration of $[B_{12}H_{12}]^{2-}$ were shown, all measurements were taken at 37°C. *d*: diameter of particle size in nanometers. d_w: diameter of particle size by the number-weighted distribution model.

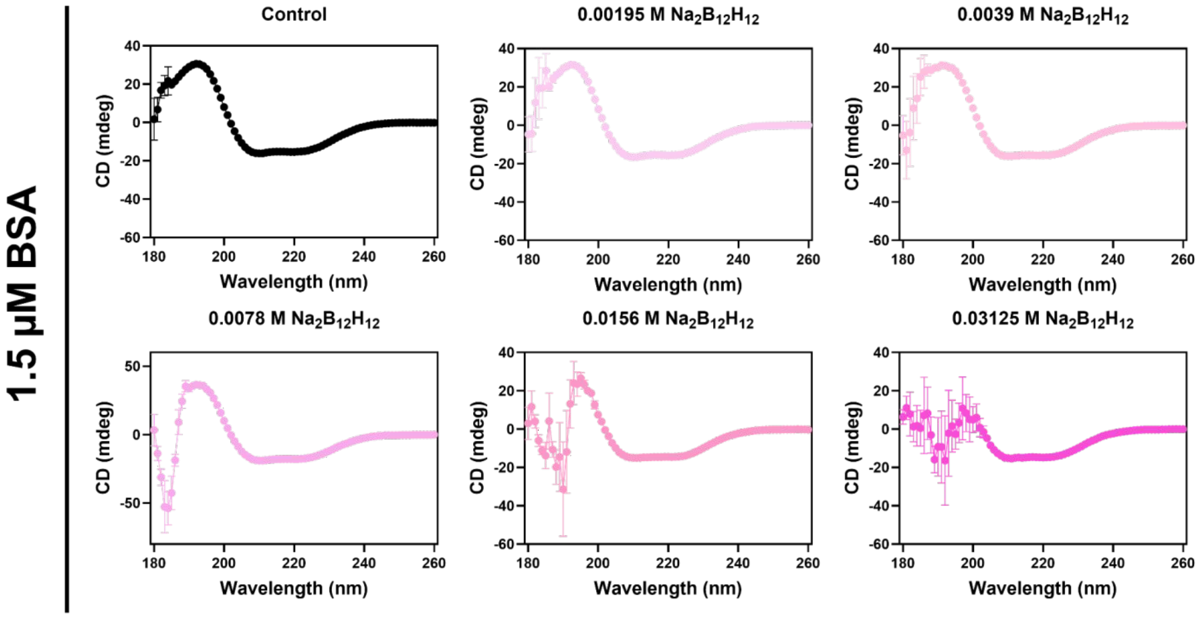

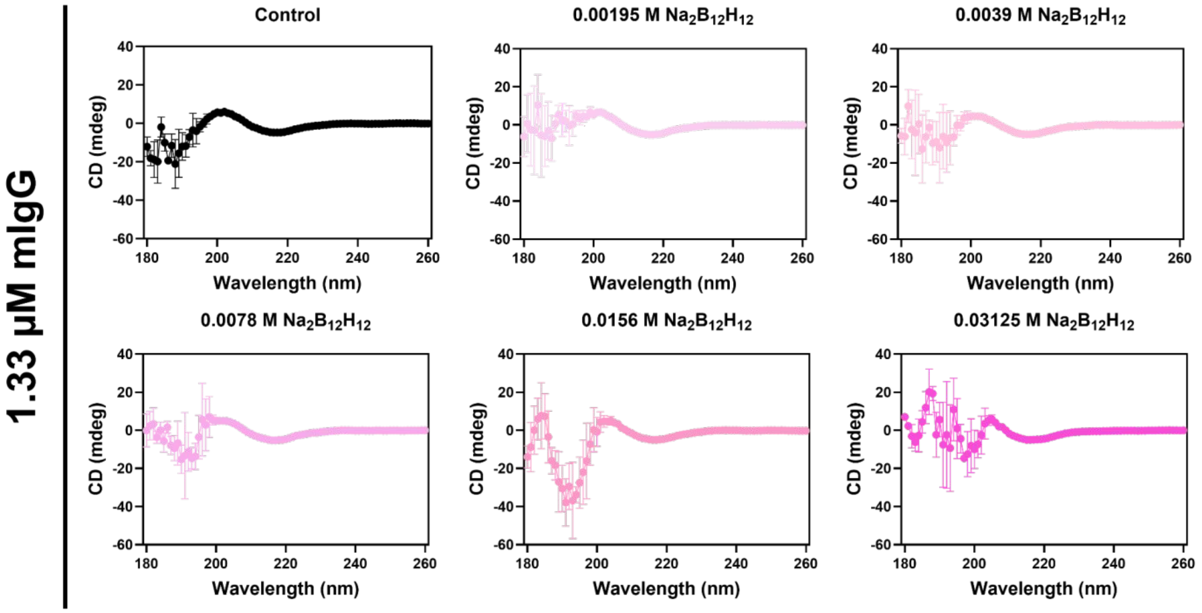

Supp Fig 2. Individual circular dichroism spectra of BSA and IgG in the presence of varying concentrations of $[B_{12}H_{12}]^{2-}$.

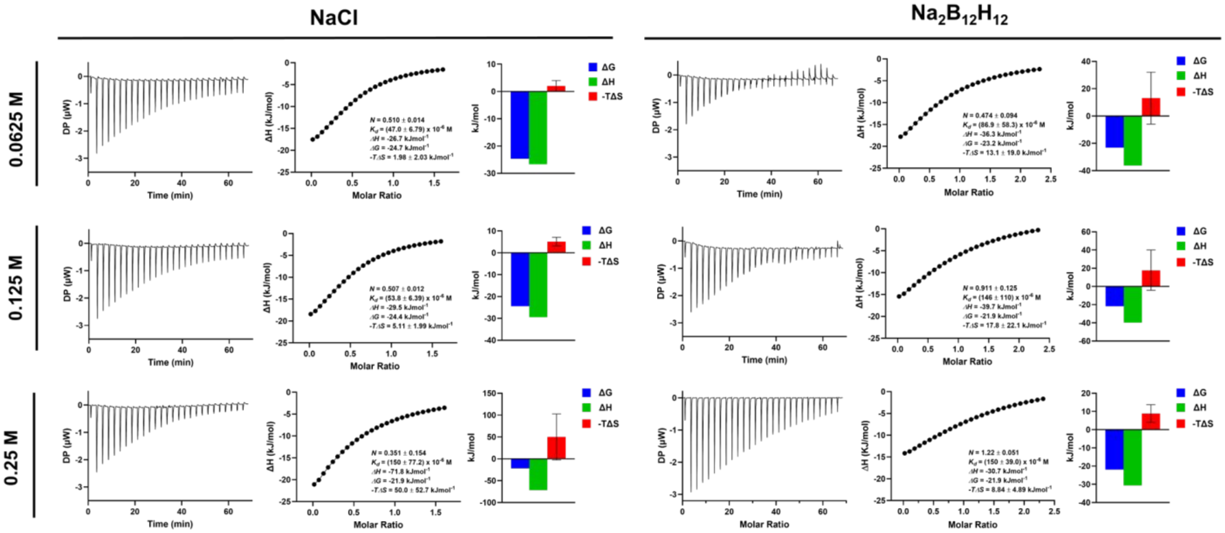

Supp Fig 3. **ITC of trypsin and ovomucoid binding reaction in the presence of various concentrations of NaCl and $Na_2B_{12}H_{12}$**. Raw tracings, fitted heat plots and thermodynamic profilings of the titration experiments were shown, with NaCl group on the left panel, and $Na_2B_{12}H_{12}$ group on the right panel.

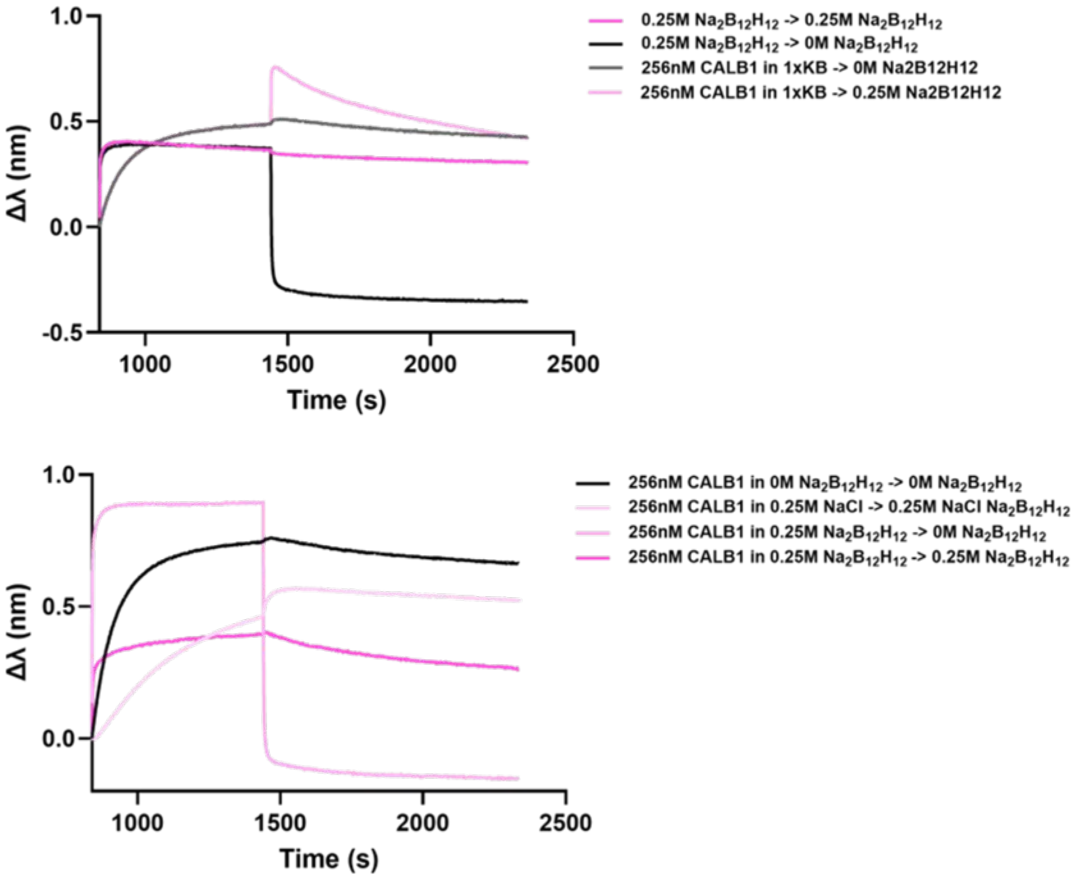

Supp Fig 4. Additional BLI data. Non-subtracted raw tracings of **Figure 4d**.

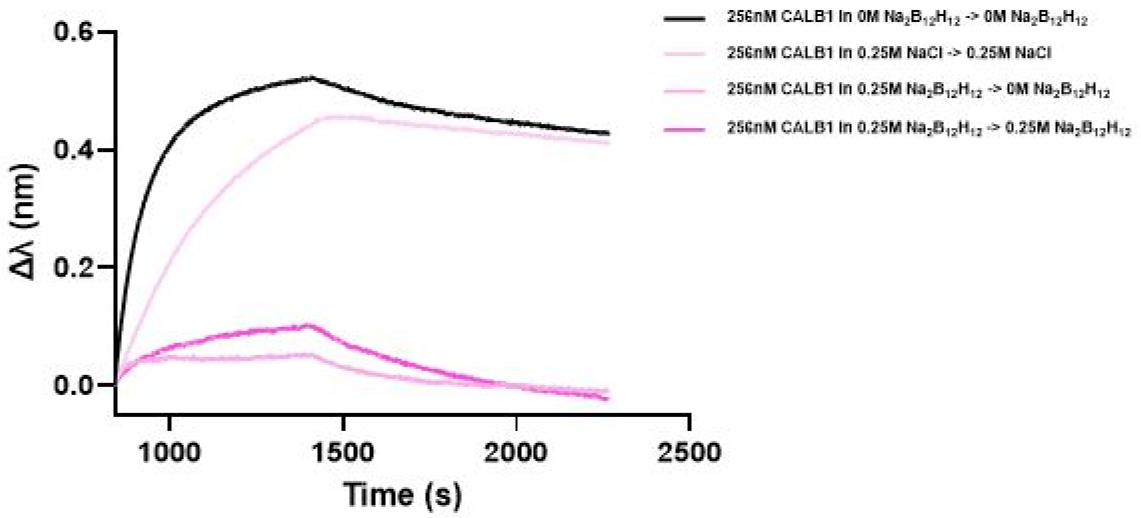

Supp Fig 5. Additional BLI data. Effect of NaCl on CALB1 binding to immobilized anti-CALB1 antibody. Tracings with the same concentration of $Na_2B_{12}H_{12}$ and control run were provided for reference.

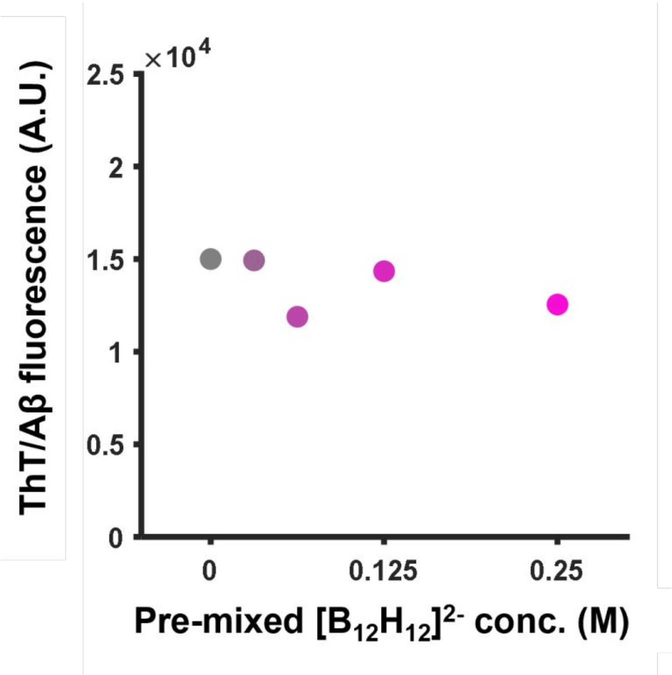

Supp Fig 6. $[B_{12}H_{12}]^{2-}$ does not alter ThT fluorescence response upon binding to Aβ fibrils. Pre-formed Aβ fibrils were first mixed with various concentrations of $[B_{12}H_{12}]^{2-}$ and then with ThT.

Supplementary Tables

Supplementary Table 1. Ranges of proteins explored in this study and their basic physicochemical properties. The predominant secondary structures were obtained from solved structures of the proteins at the protein data bank (PDB) sequence annotations. Only the predominant secondary structure was stated as the assignment to precise residues can vary for different methods and proteins are dynamic molecules.

	Approximate Molecular weight (kDa)	Isoelectric point	Predominant secondary structure
Rabbit immunoglobulin G	150	6.5-7.5 (from product insert)	β-sheets
Mouse immunoglobulin G, Isotype 1	150	6.6-7.5(102)	β-sheets
Immunoglobulin G Fab fragment	50	6.7-7.5(103)	β-sheets
Bovine serum albumin	67	4.5-4.8(104)	α-helices
Trypsin	23.3	10.1-10.5	β-sheets
Ovomucoid	28	4.1(105)	α-helices and β-sheets
Calbindin	27	4.5(106)	α-helices
Amyloid beta 1-42 peptide	4.51	5.3(107)	β-sheets
mCherry	26.7	(not found)	β-sheets
EGFP	26.9	6.2 (108)	β-sheets
BFP	26.8	(not found)	β-sheets

Supplementary Video Captions

Supp Video 1. **Boiling hen egg white in the presence of $Na_2B_{12}H_{12}$ versus NaCl**. The opacification of egg white was much delayed when it was treated with $Na_2B_{12}H_{12}$ than with NaCl.

Supplementary References

(73) Ma, L.; Pegram, L.; Record, M. T., Jr; Cui, Q. Preferential Interactions between Small Solutes and the Protein Backbone: A Computational Analysis. *Biochemistry* **2010**, *49* (9), 1954–1962.
(74) Smith, P. E. Chemical Potential Derivatives and Preferential Interaction Parameters in Biological Systems from Kirkwood-Buff Theory. *Biophys. J.* **2006**, *91* (3), 849–856.
(75) Bye, J. W.; Baxter, N. J.; Hounslow, A. M.; Falconer, R. J.; Williamson, M. P. Molecular Mechanism for the Hofmeister Effect Derived from NMR and DSC Measurements on Barnase. *ACS Omega* **2016**, *1* (4), 669–679.
(76) Srinivas, V.; Balasubramanian, D. Proline Is a Protein-Compatible Hydrotrope. *Langmuir* **1995**, *11* (7), 2830–2833.
(77) Patel, A.; Malinovska, L.; Saha, S.; Wang, J.; Alberti, S.; Krishnan, Y.; Hyman, A. A. ATP as a Biological Hydrotrope. *Science* **2017**, *356* (6339), 753–756.
(78) Pandey, M. P.; Sasidharan, S.; Raghunathan, V. A.; Khandelia, H. Molecular Mechanism of Hydrotropic Properties of GTP and ATP. *J. Phys. Chem. B* **2022**, *126* (42), 8486–8494.
(79) Mehringer, J.; Do, T.-M.; Touraud, D.; Hohenschutz, M.; Khoshsima, A.; Horinek, D.; Kunz, W. Hofmeister versus Neuberg: Is ATP Really a Biological Hydrotrope? *Cell Rep. Phys. Sci.* **2021**, *2* (2), 100343.
(80) Schreiber, G. Kinetic Studies of Protein-Protein Interactions. *Curr. Opin. Struct. Biol.* **2002**, *12* (1), 41–47.
(81) Choi, J.-M.; Serohijos, A. W. R.; Murphy, S.; Lucarelli, D.; Lofranco, L. L.; Feldman, A.; Shakhnovich, E. I. Minimalistic Predictor of Protein Binding Energy: Contribution of Solvation Factor to Protein Binding. *Biophys. J.* **2015**, *108* (4), 795–798.
(82) Neal, B. L.; Asthagiri, D.; Lenhoff, A. M. Molecular Origins of Osmotic Second Virial Coefficients of Proteins. *Biophys. J.* **1998**, *75* (5), 2469–2477.
(83) Shimizu, S. The Effect of Urea on Hydrophobic Hydration: Preferential Interaction and the Enthalpy of Transfer. *Chem. Phys. Lett.* **2011**, *517* (1-3), 76–79.
(84) Trevitt, C. R.; Yashwanth Kumar, D. R.; Fowler, N. J.; Williamson, M. P. Interactions between the Protein Barnase and Co-Solutes Studied by NMR. *Commun. Chem.* **2024**, *7* (1), 44.
(85) Hua, L.; Zhou, R.; Thirumalai, D.; Berne, B. J. Urea Denaturation by Stronger Dispersion Interactions with Proteins than Water Implies a 2-Stage Unfolding. *Proc. Natl. Acad. Sci. U. S. A.* **2008**, *105* (44), 16928–16933.
(86) Jaganade, T.; Chattopadhyay, A.; Raghunathan, S.; Priyakumar, U. D. Urea-Water Solvation of Protein Side Chain Models. *J. Mol. Liq.* **2020**, *311* (113191), 113191.
(87) Flores Jiménez, R. H.; Do Cao, M.-A.; Kim, M.; Cafiso, D. S. Osmolytes Modulate Conformational Exchange in Solvent-Exposed Regions of Membrane Proteins: Membrane Protein Conformational Exchange. *Protein Sci.* **2010**, *19* (2), 269–278.
(88) Mikhaylova, V. V.; Eronina, T. B. Effects of Osmolytes under Crowding Conditions on the Properties of Muscle Glycogen Phosphorylase B. *Biochimie* **2024**, *220*, 48–57.
(89) Panuszko, A.; Bruździak, P.; Kaczkowska, E.; Stangret, J. General Mechanism of Osmolytes' Influence on Protein Stability Irrespective of the Type of Osmolyte Cosolvent. *J. Phys. Chem. B* **2016**, *120* (43), 11159–11169.
(90) Janin, J. The Kinetics of Protein-Protein Recognition. *Proteins* **1997**, *28* (2), 153–161.

(91) Alberty, R. A.; Hammes, G. G. Application of the Theory of Diffusion-Controlled Reactions to Enzyme Kinetics. *J. Phys. Chem.* **1958**, *62* (2), 154–159.
(92) Jones, G.; Dole, M. The Viscosity of Aqueous Solutions of Strong Electrolytes with Special Reference to Barium Chloride. *J. Am. Chem. Soc.* **1929**, *51* (10), 2950–2964.
(93) Jenkins, H. D. B.; Marcus, Y. Viscosity B-Coefficients of Ions in Solution. *Chem. Rev.* **1995**, *95* (8), 2695–2724.
(94) Onsager, L. Electric Moments of Molecules in Liquids. *J. Am. Chem. Soc.* **1936**, *58* (8), 1486–1493.
(95) Kirkwood, J. G. The Dielectric Polarization of Polar Liquids. *J. Chem. Phys.* **1939**, *7* (10), 911–919.
(96) Bordewijk, P. On the Derivation of the Kirkwood-Fröhlich Equation. *Physica* **1973**, *69* (2), 422–432.
(97) Wang, S.; Walker-Gibbons, R.; Watkins, B.; Flynn, M.; Krishnan, M. A Charge-Dependent Long-Ranged Force Drives Tailored Assembly of Matter in Solution. *Nat. Nanotechnol.* **2024**, *19* (4), 485–493.
(98) Shubina, E. S.; Bakhmutova, E. V.; Filin, A. M.; Sivaev, I. B.; Teplitskaya, L. N.; Chistyakov, A. L.; Stankevich, I. V.; Bakhmutov, V. I.; Bregadze, V. I.; Epstein, L. M. Dihydrogen Bonding of Decahydro-Closo-decaborate(2−) and Dodecahydro-Closo-dodecaborate(2−) Anions with Proton Donors: Experimental and Theoretical Investigation. *J. Organomet. Chem.* **2002**, *657* (1-2), 155–162.
(99) Jiang, Y.; Yuan, Q.; Cao, W.; Hu, Z.; Yang, Y.; Zhong, C.; Yang, T.; Sun, H.; Wang, X.-B.; Sun, Z. Unraveling Hydridic-to-Protonic Dihydrogen Bond Predominance in Monohydrated Dodecaborate Clusters. *Chem. Sci.* **2022**, *13* (34), 9855–9860.
(100) Bauduin, P.; Prevost, S.; Farràs, P.; Teixidor, F.; Diat, O.; Zemb, T. A Theta-Shaped Amphiphilic Cobaltabisdicarbollide Anion: Transition from Monolayer Vesicles to Micelles. *Angew. Chem. Int. Ed Engl.* **2011**, *50* (23), 5298–5300.
(101) Fink, K.; Cebula, J.; Tošner, Z.; Psurski, M.; Uchman, M.; Goszczyński, T. M. Cobalt Bis(dicarbollide) Is a DNA-Neutral Pharmacophore. *Dalton Trans.* **2023**, *52* (30), 10338–10347.
(102) Danielsson, A.; Ljunglöf, A.; Lindblom, H. One-Step Purification of Monoclonal IgG Antibodies from Mouse Ascites. An Evaluation of Different Adsorption Techniques Using High Performance Liquid Chromatography. *J. Immunol. Methods* **1988**, *115* (1), 79–88.
(103) Coleman, L.; Mahler, S. M. Purification of Fab Fragments from a Monoclonal Antibody Papain Digest by Gradiflow Electrophoresis. *Protein Expr. Purif.* **2003**, *32* (2), 246–251.
(104) Raghuwanshi, V. S.; Yu, B.; Browne, C.; Garnier, G. Reversible pH Responsive Bovine Serum Albumin Hydrogel Sponge Nanolayer. *Front. Bioeng. Biotechnol.* **2020**, *8*, 573.
(105) Kido, J.; Matsumoto, T. Attenuated Allergenic Activity of Ovomucoid after Electrolysis. *Allergy Asthma Immunol. Res.* **2015**, *7* (6), 599–604.
(106) Gross, M. D.; Kumar, R.; Hunziker, W. Expression in Escherichia Coli of Full-Length and Mutant Rat Brain Calbindin D28. Comparison with the Purified Native Protein. *J. Biol. Chem.* **1988**, *263* (28), 14426–14432.
(107) Roche, J.; Shen, Y.; Lee, J. H.; Ying, J.; Bax, A. Monomeric Aβ(1-40) and Aβ(1-42) Peptides in Solution Adopt Very Similar Ramachandran Map Distributions That Closely Resemble Random Coil. *Biochemistry* **2016**, *55* (5), 762–775.

(108) Gurunathan, S.; Woong Han, J.; Kim, E.; Kwon, D.-N.; Park, J.-K.; Kim, J.-H. Enhanced Green Fluorescent Protein-Mediated Synthesis of Biocompatible Graphene. *J. Nanobiotechnology* **2014**, *12* (1), 41.
